# 6PPD and 6PPD-Q Induce Mitochondrial Dysfunction in *Saccharomyces cerevisiae*

**DOI:** 10.3390/ijms27177739

**Published:** 2026-08-29

**Authors:** Emma Braun, Mileah Metcalf, Kyoungtae Kim

**Affiliations:** Department of Biology, Missouri State University, 901 S National, Springfield, MO 65897, USA; eb2872s@missouristate.edu (E.B.); mcm68s@missouristate.edu (M.M.)

**Keywords:** 6PPD, 6PPD-Quinone, tire-wear particles, toxicant, mitochondria, metabolism

## Abstract

N-(1,3-dimethylbutyl)-N′phenyl-p-phenylenediamine, more commonly known as 6PPD, is a tire antiozonant used to prevent rubber oxidation and degradation. When 6PPD reacts with ozone, it becomes 6PPD-Quinone (6PPD-Q). Stormwater road runoff facilitates the transport of phenylenediamines (PPDs) into the environment. In recent years, 6PPD-Q has been identified as an acute environmental toxicant causing major disruptions to aquatic life. To aid in the growing body of knowledge on the mechanism of action of PPD and potentially support environmental regulations, this study aims to elucidate the toxic effects of 6PPD and 6PPD-Q in *Saccharomyces cerevisiae*. To accomplish this, a growth assay, RNA sequencing, mitochondrial staining, and RT-qPCR were performed. The results suggest that 6PPD and 6PPD-Q impact growth and gene regulation by dysregulating Complex III, IV, and V of the oxidative phosphorylation pathway. Additionally, 6PPD and 6PPD-Q are, seemingly, agglomerated at different pHs and in various media. The agglomeration of 6PPD-Q may inhibit cellular uptake, leading to fewer discernible toxic effects. Taken together, we can posit that 6PPD and 6PPD-Q both induce oxidative stress upon exposure to budding yeast, but 6PPD seems to be more toxic than 6PPD-Q.

## 1. Introduction

6PPD, N-(1,3-dimethylbutyl)-N′phenyl-p-phenylenediamine, was developed and introduced in the 1960s to combat the breakdown of rubber tires in the presence of ozone. Ozone specifically targets the carbon–carbon double bond in the rubber polymer chain through a process called ozonolysis, which weakens tire integrity [1]. Since its rise to popularity, 6PPD has been mainly used to prolong the lifetime of vehicle tires but has also been integrated into other products like asphalt, turf, sneaker soles, door mats, and rubber gloves [2,3,4]. 6PPD, with an approximate half-life of 24 h, helps mitigate the degradation of rubber by creating a protective layer and reacting rapidly with ozone [5]. Through this reaction, the main transformation product (TP) 6PPD-Quinone (6PPD-Q) is formed.

Both 6PPD and 6PPD-Q can readily enter the environment in the form of tire-wear particles (TWPs). TWPs fall into the category of microplastics, which have been detected in air particulate matter (PM), soil, and road runoff [6,7,8]. The detection of 6PPD-Q and additional TWPs in stormwater road runoff has become a major concern for urban watersheds [9]. In 2021, 6PPD-Q was identified in the U.S. Pacific Northwest as the cause of the acute mortality of coho salmon (*Oncorhynchus kisutch*) as they returned to urban watersheds to spawn [10]. This phenomenon is often referred to as urban runoff mortality syndrome (URMS) [11,12,13]. Common environmental concentrations of 6PPD-Q from stormwater road runoff range from 0.1 µg/L to 10 µg/L [11,14,15]. Given that the original LC_50_ for coho salmon is 0.8 µg/L, the influx of 6PPD-Q into the environment raises concerns [10].

To combat the alarming lethality of 6PPD-Q, global leaders, including the United States, Canada, and the European Union, are drafting regulations for PPDs [16,17,18]. Until these are implemented, TWPs will continue to impact the environment and, potentially, human health. Presently, there are no alternatives to 6PPD being used in tire production. There are alternative PPDs in development, including 7PPD, 77PD, IPPD, and CCPD [19,20]. IPPD may be a promising alternative because of its low biological activity. However, there are indications that 7PPD-Q and 77PD-Q may have the same toxic effects on aquatic life and the same potential reproductive health repercussions as 6PPD [20]. Additionally, non-PPD alternatives, Durazone (PPDTZ), graphene, octyl gallate, 1,2-dihydro-2,2,4-trimethylquinoline (TMQ), and amine-functionalized lignin, are also being investigated [21,22,23,24,25]. While these alternatives hold promise, continuing research elucidating 6PPD and 6PPD-Q’s toxic effects is pertinent.

Expanding on aquatic organism toxicology, other salmonoid model organisms have been used to investigate 6PPD-Q’s toxic effects. Among salmonoid species, Rainbow Trout (*Oncorhynchus mykiss*) (72 h LC_50_ = 1 µg/L 6PPD-Q), Brook Trout (*Salvelinus fontinalis*) (24 h LC_50_ = 0.59 µg/L 6PPD-Q), and White Spotted Char (Salvelinus *leucomaenis*) (24 h LC_50_ = 0.51 µg/L 6PPD-Q) exhibit acute toxicity to 6PPD-Q [26,27,28]. Other salmonoid models, including Arctic Char (*Salvelinus alpinus*), White Sturgeon (*Sinosturio transmontanus*), Brown Trout (*Salmo trutta*), Atlantic salmon (*Salmo salar*), Chinook (*Oncorhynchus tshawytscha*), Sockeye salmon (*Oncorhynchus nerka*), Southern Asian Dolly Varden (*Salvelinus curilus*), and Landlocked Masu Salmon (*Oncorhynchus masou formosanus*) are more tolerant to environmental concentrations ranging from 3.8 µg/L to 50 µg/L 6PPD-Q [26,27,28,29,30].

To further understand 6PPD-Q’s mechanism of action, zebrafish (*Danio rerio*), Caenorhabditis Elegans (*C. elegans*), mice, and mammalian cell lines have been employed as toxicology models. While the focus has been on 6PPD-Q, 6PPD has also been implemented in many studies for toxicology comparisons. Recent studies have revealed several organ, reproductive, and developmental toxicities in the presence of 6PPD and 6PPD-Q. In zebrafish, 10 µg/L and 25 µg/L of 6PPD and 6PPD-Q, respectively, induced morphological malformities, behavioral changes, and cardiotoxicity [31]. *C. elegans* experienced reduced reproduction, decreased mitochondrial function, and impaired development when exposed to 0.5 mM 6PPD [32]. Additionally, at lower concentrations, 1–100 µg/L 6PPD-Q, *C. elegans* also exhibited reproductive toxicity [33]. Both male and female mice have had 6PPD and 6PPD-Q detected in their urine output and in the female placenta [34]. Likewise, 6PPD and 6PPD-Q have also been reported to induce hepatotoxicity in mice [35,36]. 6PPD-Q has also been linked to various dysfunctions in human ovarian granulosa (KGN), non-small cell lung cancer (NSCLC), the human proto-hepatocyte model (L02), and the human hepatocellular carcinoma cell line (HepG2), among many others [37,38,39]. As the list of model organisms expands, there are still substantial gaps to fill.

Currently, there are no papers investigating the toxic effects of PPDs on single-celled eukaryotic organisms. This work focuses on using *Saccharomyces cerevisiae*, budding yeast, as a model organism. Budding yeasts are ubiquitous in the environment, being closely associated with plants and soil, and having a fully sequenced genome with conserved DNA regions containing rRNA, metabolic, and histone protein genes [40]. Because of their versatile nature, yeasts can be used as an indicator for environmental and human health. To add to the growing knowledge base surrounding 6PPD and 6PPD-Q, and potentially contribute to the development of regulations, we explored the effects of both PPDs on *Saccharomyces cerevisiae*. We hypothesized that 6PPD and 6PPD-Q would affect budding yeast metabolism, specifically targeting energy production via the oxidative phosphorylation process. This study also aims to compare the toxic effects of 6PPD and 6PPD-Q on yeast growth, gene regulation, and mitochondrial function. Additionally, both PPDs may demonstrate the ability to agglomerate in buffers at various pH’s, and in common growth mediums.

## 2. Results

### 2.1. Yeast Growth Assay

**Figure 1:**
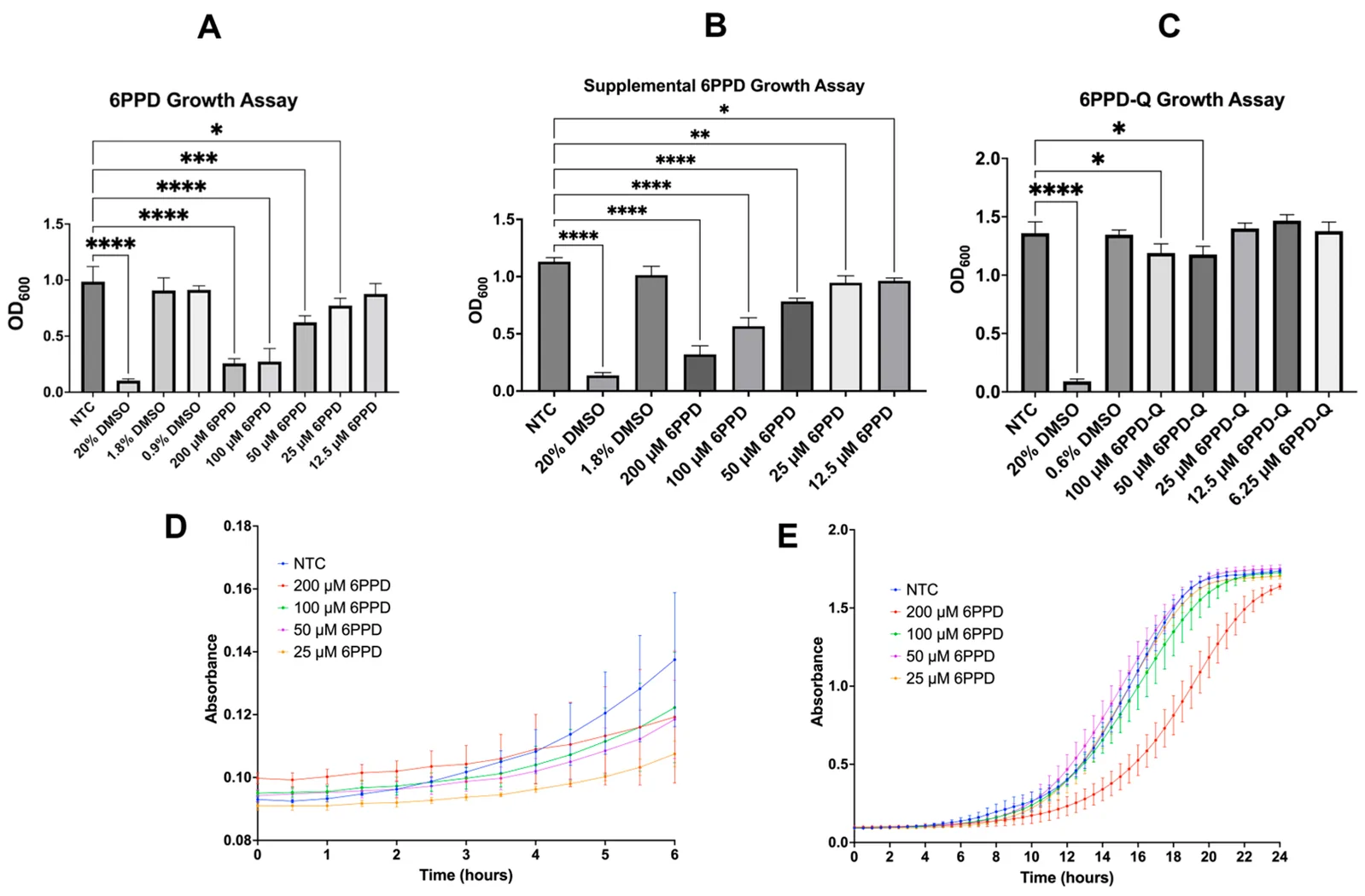
Yeast growth assay to determine 6PPD and 6PPD-Q’s effect on yeast proliferation. (**A**) 6-h growth assay for 6PPD. (**B**) Supplemental 6-h growth assay for 6PPD with constant concentrations of 1.8% DMSO per sample. (**C**) 6-h growth assay for 6PPD-Q. (**D**) 6-h kinetic growth curve. (**E**) 24-h kinetic growth curve. Data are expressed as mean ± SD and * *p* < 0.05, ** *p* < 0.01, *** *p* < 0.001, and **** *p* < 0.0001.

The effect of 6PPD and 6PPD-Q on yeast proliferation was assessed with a growth assay and interpreted using one-way ANOVA. Background absorbance of 6PPD-Q was subtracted from each treatment group. Upon treatment of 6PPD, there was a dose-dependent decrease in viability ranging from the highest concentration, 200 µM, down to 25 µM (Figure 1A). From this data set, the IC_50_ value of 56 µM 6PPD was calculated. To ensure the DMSO solvent concentration did not impact viability, a secondary growth assay with constant 1.8% DMSO in each treatment was tested (Figure 1B). The same trend of viability was seen, ensuring DMSO had no impact on the decrease in viability. Because of previously reported instances of 6PPD-Q being more toxic than 6PPD in coho salmon, *Chlorella vulgaris*, and black spotted frogs, the concentration range was scaled down in anticipation of similar results [41,42,43]. However, when exposed to 6PPD-Q, there was only a slight decrease in yeast viability at the two highest concentrations, 100 and 50 µM 6PPD-Q (Figure 1C). Additionally, we investigated yeast growth using a kinetic growth assay for 6PPD. Consistent with our end-point assay at 6 h, there was a slight delay in the growth of the treated groups starting at hour 5 (Figure 1D). Over a 24-h period, all treated groups besides 200 µM 6PPD seemed to recover and reach a log-phase growth rate similar to the non-treated group (Figure 1E).

### 2.2. 6PPD and 6PPD-Q Agglomeration in Growth Media

**Figure 2:**
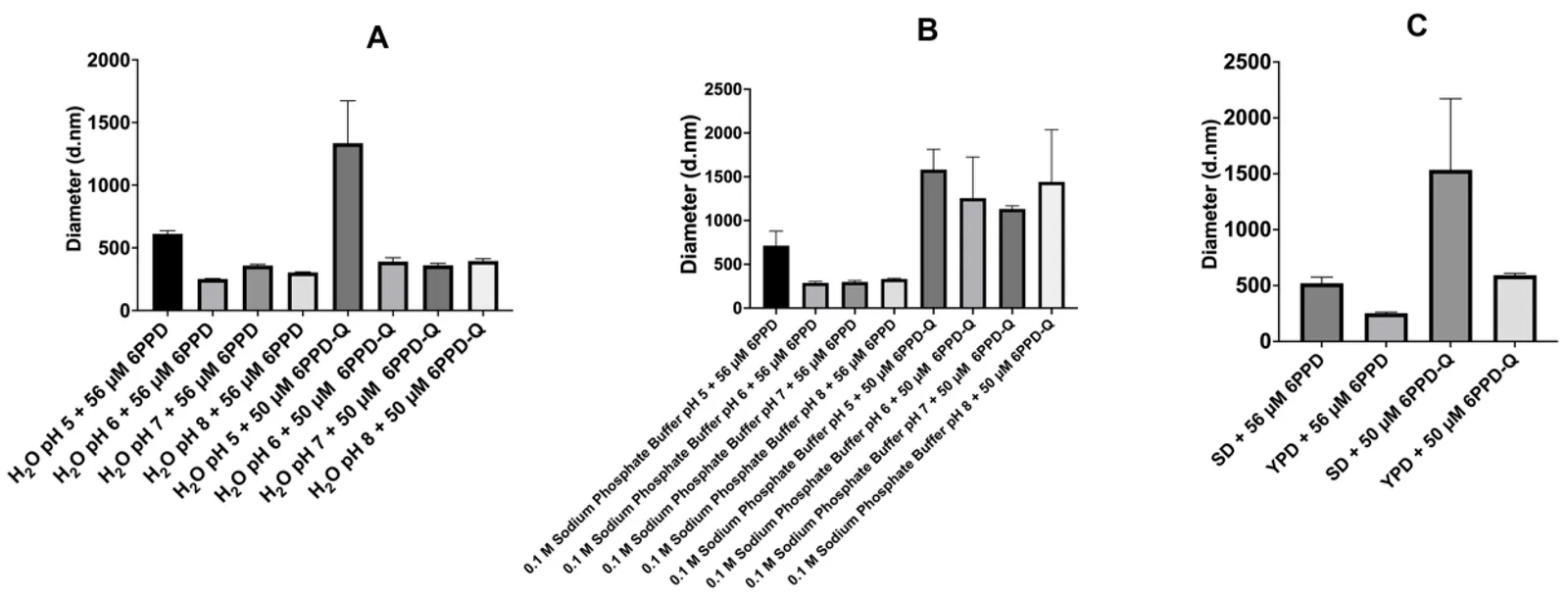
Dynamic light scattering (DLS) for 6PPD and 6PPD-Q showing agglomeration in different media. (**A**) 6PPD and 6PPD-Q size variance in water at pH 5, 6, 7, and 8. (**B**) 6PPD and 6PPD-Q size variance in sodium phosphate buffer at pH 5, 6, 7, and 8. (**C**) 6PPD and 6PPD-Q size variance in synthetic defined (SD) media, and yeast peptone dextrose (YPD) media. Data are expressed as mean ± SD.

From the growth assay, we originally noticed an increase in growth after treatment with 6PPD-Q, which was corrected by subtracting background absorbance. Since 6PPD-Q seemingly agglomerated in YPD, the IC_50_ of 6PPD (56 mM) and the minimal effective concentration of 6PPD-Q (50 µM) were analyzed in deionized water with a range of pHs. At pH 5, 6PPD (611.9 ± 24.25 nm) and 6PPD-Q (1335 ± 338.4 nm) exhibited the largest diameter (Figure 2A). At all other pHs, the average diameter for both 6PPD and 6PPD-Q stayed within the range of 250 to 400 nm. A similar trend was seen with 6PPD (713.2 ± 166.8 nm) in pH 5 sodium phosphate buffer, while at all other pHs the average size stayed around 300 nm (Figure 2B). Despite the pH changing, 6PPD-Q exhibited a large diameter in sodium phosphate buffer, with the largest average diameter being 1581 ± 231.9 nm at pH 5 (Figure 2B).

The diameter of both PPDs was investigated in SD and YPD growth media. 6PPD had a slightly larger diameter in SD (520.6 ± 54.69 nm) compared to YPD (252.5 ± 9.546 nm) (Figure 2C). Comparatively, there was a similar trend, with 6PPD-Q having a larger diameter in SD (1535 ± 636.7 nm), as opposed to YPD (592.1 ± 1 6.62 nm) (Figure 2C). Lastly, we measured the overall surface charge of both PPDs in YPD media (Table 1). YPD alone compared to YPD with 6PPD, and these had very similar average charges: −8.08 and −8.33 mV, respectively. In comparison, YPD with 6PPD-Q exhibited a more negative overall charge of −13.2 mV. YPD typically has a slightly acidic pH of around 5.5 to 6.5, with many free ions. The free ions could be closely associated with the quinone functional groups or amine functional groups of 6PPD-Q, causing the overall charge to be negative. This could also be a possible explanation for the fluctuation of PPD sizes seen in the DLS data.

**Figure 3:**
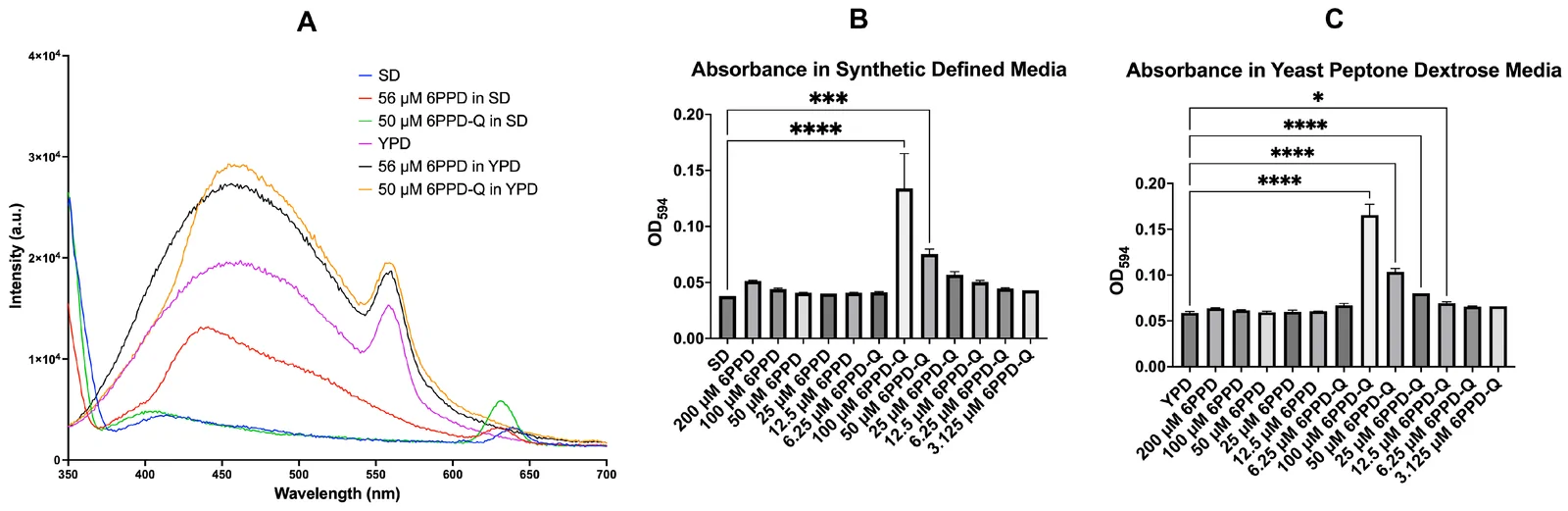
Fluorescence and absorbance of 6PPD and 6PPD-Q in YPD and SD media. (**A**) Fluorescence data for 56 µM 6PPD and 50 µM 6PPD-Q in SD and YPD media. (**B**) Absorbance (594 nm) of 6PPD and 6PPD-Q at different concentrations in SD media. (**C**) Absorbance (594 nm) of 6PPD and 6PPD-Q at different concentrations in YPD media. Data are expressed as mean ± SD and * *p* < 0.05, *** *p* < 0.001, and **** *p* < 0.0001.

Because of the potential agglomeration reported, the intrinsic fluorescence and absorbance in SD and YPD were measured for both PPDs. In SD media, 6PPD-Q showed similar fluorescence intensity to SD, with a small peak at 630 nm, while 6PPD had a large peak of fluorescence at 440 nm (Figure 3A). In YPD media, both 6PPD and 6PPD-Q exhibited increased fluorescence, with peak absorbance ranging from 450 to 460 nm and from 550 to 570 nm (Figure 3A). 6PPD did not have any background fluorescence in SD or YPD media (Figure 3B, C). In SD media, 6PPD-Q had high background absorbance at concentrations 100 and 50 µM (Figure 3B). Comparatively, in YPD media, 6PPD-Q exhibited high background absorbance at concentrations 100, 50, 25, and 12.5 µM (Figure 3C). The increased absorbance is likely attributed to agglomeration and was considered when analyzing the growth assay data.

### 2.3. RNA Sequencing Analysis

**Figure 4:**
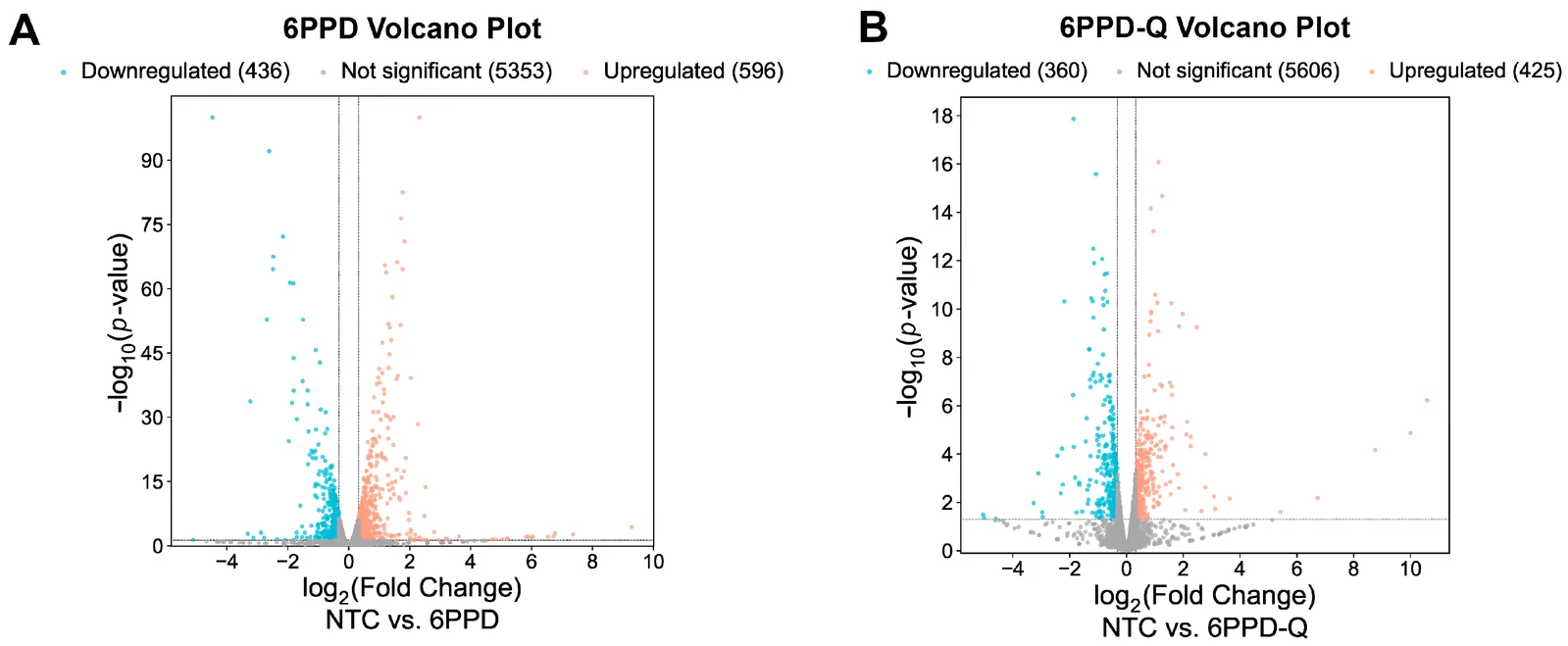
Up- and downregulated gene transcripts after 4.5-h exposure to 6PPD and 6PPD-Q. (**A**) Volcano plot for 6PPD showing 596 upregulated and 436 downregulated genes with a fold change of ±1.25 and a *p*-value < 0.05. (**B**) Volcano plot for 6PPD-Q showing 425 upregulated and 360 downregulated genes with a ±1.25-fold change and a *p*-value < 0.05. The fold change was converted to −log_2_, and the *p*-value was converted to −log_10_. Blue dots represent downregulation, and red dots represent upregulation. Volcano plots were generated using SRplot.

RNA sequencing was performed to elucidate the underlying genes associated with the decrease in growth and metabolic function. Total RNA from non-treated and treated samples was extracted and converted into cDNA. cDNA samples were shipped to Novogene for analysis. After sequencing, a total of 141,646,905 (91.6%) reads were mapped for the control group, 87,931,009 (91.0%) reads were mapped for the 6PPD treatment, and 149,228,306 (86.4%) reads were mapped for the 6PPD-Q treatment. Treatment of 6PPD caused upregulation of 596 genes and downregulation of 436 genes (Figure 4A). The treatment of 6PPD-Q yielded different results, with 425 upregulated and 360 downregulated genes (Figure 4B).

**Figure 5:**
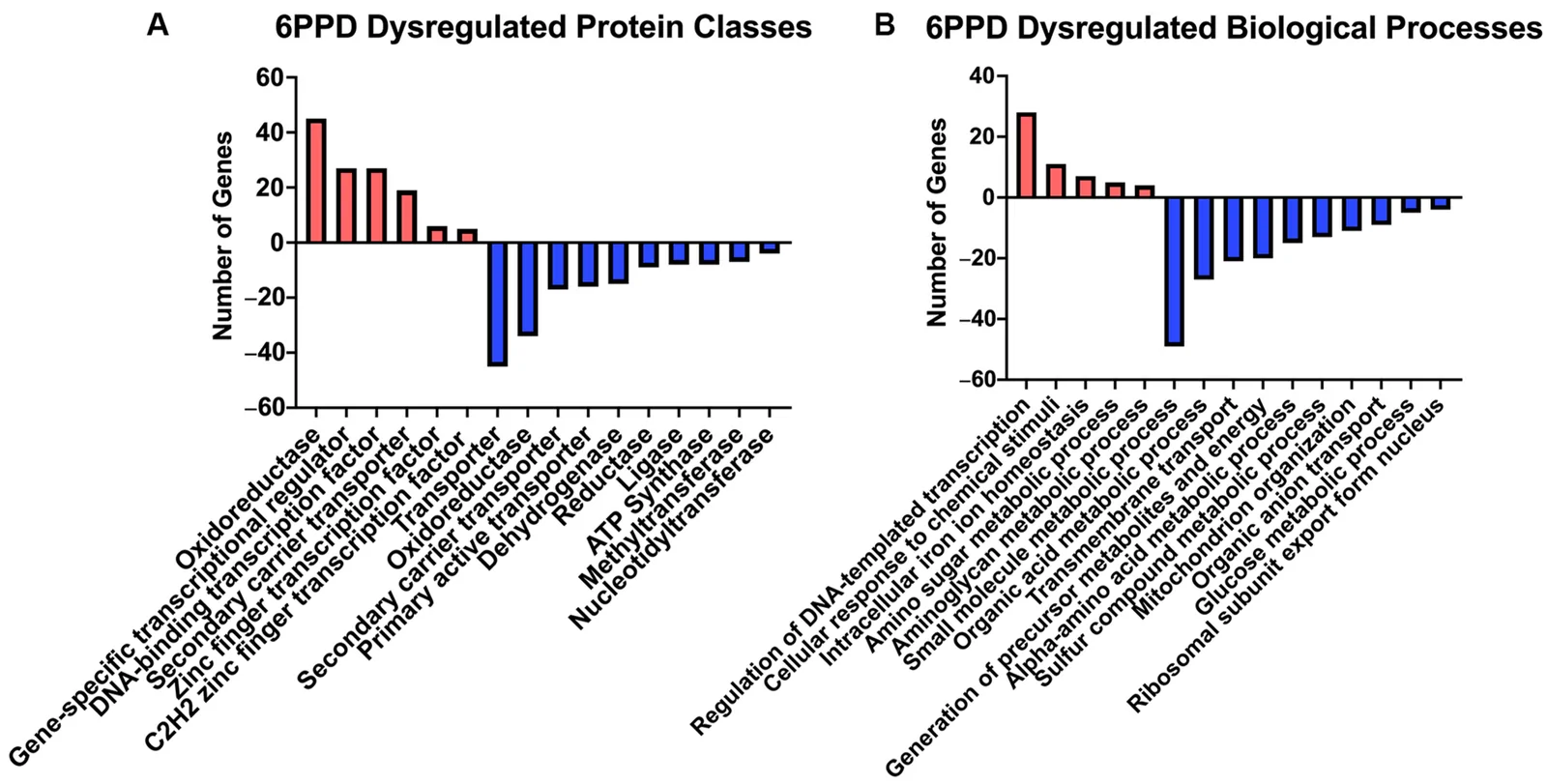
Bar graphs representing genes associated with protein classes and biological processes that were differentially affected upon exposure to 6PPD. (**A**) 6PPD dysregulated protein class genes. (**B**) 6PPD dysregulated biological process genes. Categories of genes were determined using Panther Gene Ontology with a Fold Enrichment ≥ 2.00 and FDR *p*-value < 0.05. Each bar shows the total number of dysregulated genes within each category. Red bars indicate upregulated categories, and blue bars indicate downregulated categories.

The total number of up- and downregulated genes was categorized into protein classes using Panther Gene Ontology. Based on our GO analysis, the enriched protein classes for 6PPD upregulated genes are oxidoreductase, gene-specific transcriptional regulator, DNA-binding transcription factor, zinc finger transcription factor, and C2H2 zinc finger transcription factor (Figure 5A). The enriched protein classes for 6PPD downregulated genes include transporter, oxidoreductase, secondary carrier transporter, primary active transporter, dehydrogenase, reductase, ligase, ATP synthase, methyltransferase, and nucleotidyltransferase (Figure 5A). A similar GO analysis was used to categorize genes into biological processes. There were 5 enriched biological processes with genes upregulated by 6PPD: regulation of DNA-templated transcription, cellular response to chemical stimuli, intracellular iron ion homeostasis, amino sugar metabolic process, and aminoglycan metabolic process (Figure 5B). Comparatively, there were 10 enriched biological protein classes for 6PPD downregulated genes: small molecule metabolic process, organic acid metabolic process, transmembrane transport process, generation of precursor metabolites and energy, alpha-amino acid metabolic process, sulfur compound metabolic process, mitochondrion organization, organic ion transport, glucose metabolic process, and ribosomal subunit export from nucleus (Figure 5B).

**Figure 6:**
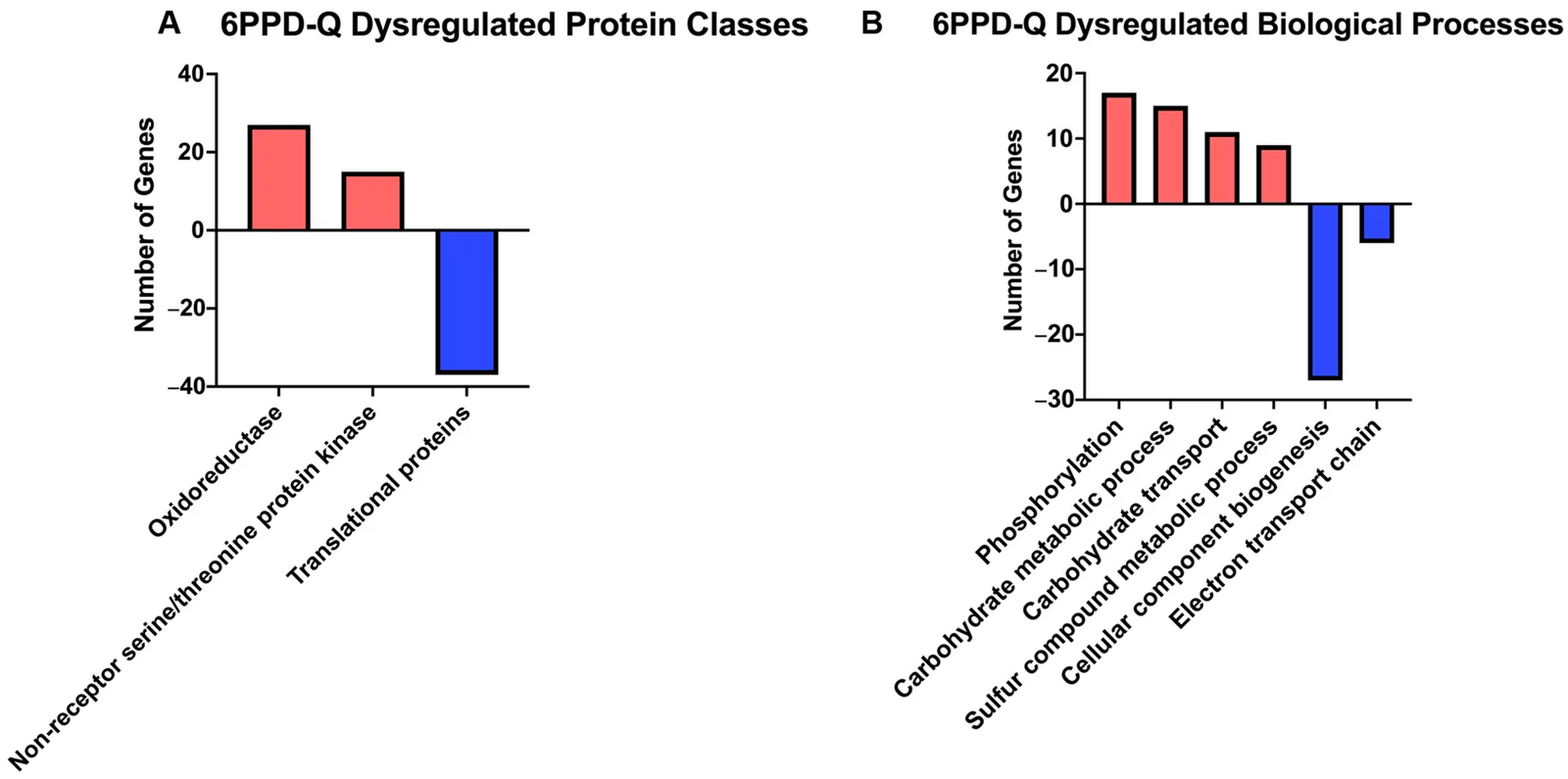
Bar graphs representing genes associated with protein classes and biological processes that were differentially affected upon exposure to 6PPD-Q. (**A**) 6PPD-Q dysregulated protein class genes. (**B**) 6PPD-Q dysregulated biological process genes. Categories of genes were determined using Panther Gene Ontology with a Fold Enrichment ≥ 2.00 and FDR *p-*value < 0.05. Each bar shows the total number of dysregulated genes within each category. Red bars indicate upregulated categories, and blue bars indicate downregulated categories.

A similar analysis was done for 6PPD-Q up- and downregulated genes. Based on our GO analysis, the enriched protein classes for 6PPD-Q upregulated genes are oxidoreductase and non-receptor serine/threonine protein kinase (Figure 6A). There was only one enriched protein class for 6PPD-Q downregulated genes: translational proteins (Figure 6A). There were four enriched biological processes with genes upregulated by 6PPD-Q: phosphorylation, carbohydrate metabolic process, carbohydrate transport, and sulfur compound metabolic process (Figure 6B). Comparatively, there were only two enriched biological protein classes for 6PPD-Q downregulated genes: cellular component biogenesis and electron transport chain (Figure 6B).

**Figure 7:**
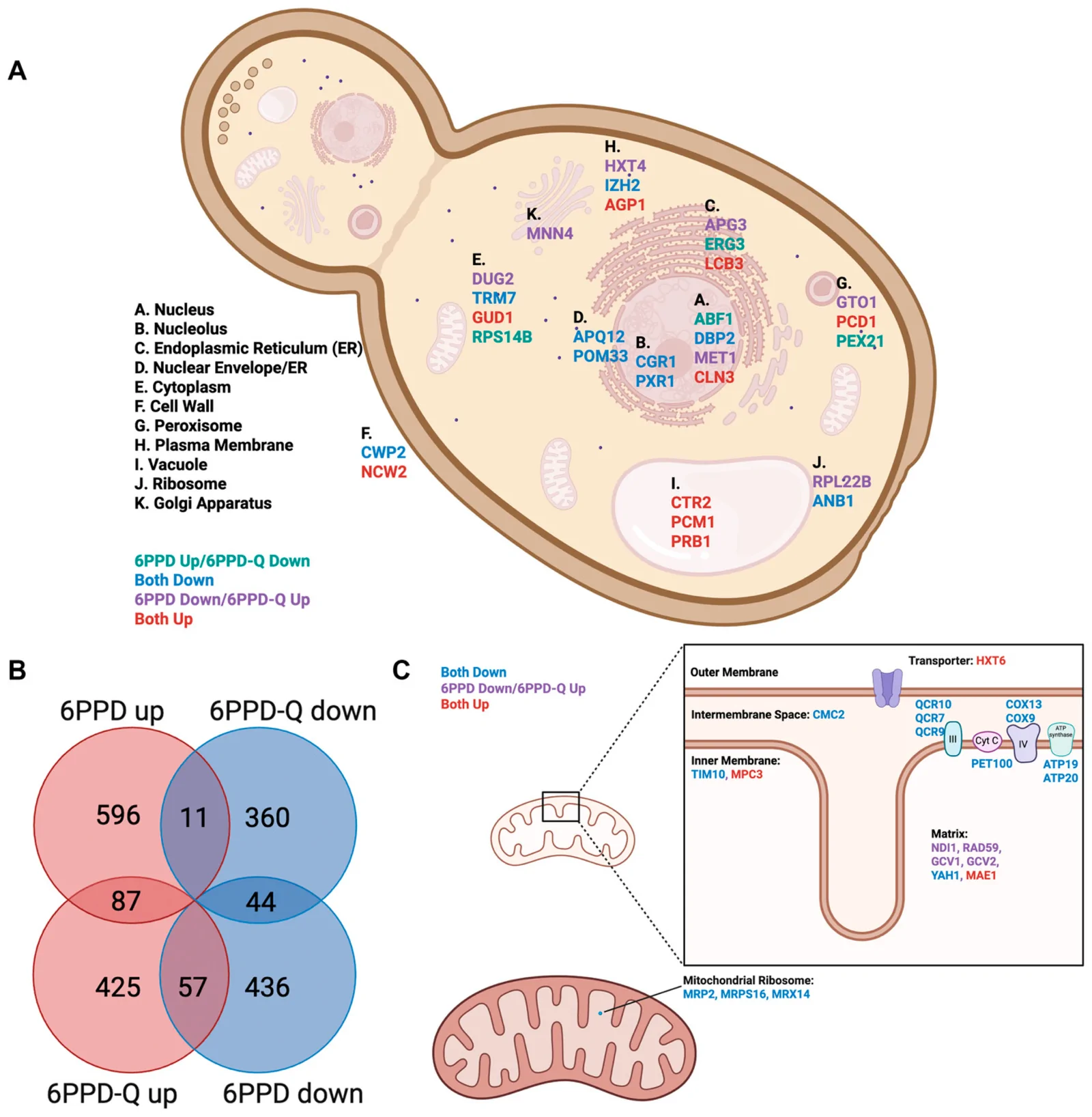
Models showing DEGs associated with yeast organelles. (**A**) DEGs associated with common organelles. (**B**) Ven Diagram of up- and downregulated genes after 6PPD of 6PPD-Q exposure. Overlapping areas represent shared genes between the two PPDs. (**C**) DEGs associated with the mitochondria. Upregulated genes are shown in red, downregulated genes are shown in blue, 6PPD up- and 6PPD-Q downregulated genes are shown in green, and 6PPD down- and 6PPD-Q upregulated genes are shown in purple. Created using Biorender.com.

To create a full picture of how 6PPD and 6PPD-Q were affecting yeast organelles, the up- and downregulated genes for each treatment, as well as differentially expressed genes (DEGs) between the two PPDs, were made into a yeast model (Figure 7A). Seemingly, almost every major organelle present in budding yeast had at least one gene that was affected by 6PPD or 6PPD-Q. Of the total number of genes that were dysregulated, several genes overlapped between the treatments (Figure 7B). Within the overlapping genes, there were many implicated in the mitochondria, like *QCR10* (Figure 7C). The gene *QCR10* encodes for a subunit of the cytochrome bc1 complex, and when knocked out, reduces ubiquinol-cytochrome c oxidoreductase activity by 40% [44]. In both treatment groups, *QCR10* was downregulated (6PPD fold change −1.425, *p*-value 0.000206; 6PPD-Q fold change −1.507, *p*-value 0.000548) (Supplemental Table S1).

The gene *MPC3* was upregulated for both 6PPD (fold change 3.15, *p*-value 5.32 × 10^−12^) and 6PPD-Q (fold change 1.85, *p*-value 0.000587) (Figure 7C, Supplemental Table S2). Mpc3 protein complexes maintain cellular homeostasis and metabolism by transporting pyruvate into the mitochondrial matrix [45]. Under oxidative stress conditions, Mpc3 is typically upregulated, making it a viable stress response marker [46]. A different transport protein encoded by *HTX6* was also upregulated for both PPDs (6PPD fold change 1.56, *p*-value 9.73 × 10^−5^; 6PPD-Q fold change 2.18, *p*-value 6.60 × 10^−6^) (Supplemental Table S2). Hxt6 is a high-affinity hexose transporter located on the outer membrane of the mitochondria [47]. When placed under high-pressure stress conditions, *HXT6* is typically upregulated [48]. The overexpression of *MPC3* and *HXT6* indicates a possible stress response to 6PPD and 6PPD-Q.

Between the two PPDs, the mechanism of action seems to differ. In total, there are 57 genes that were downregulated for 6PPD but upregulated for 6PPD-Q. For example, *GSY1,2*, *NDI1*, and *RAD59* have been linked to mitochondrial functions. *GSY1* (6PPD fold change −1.48 and 6PPD-Q fold change 4.30) and *GSY2* (6PPD fold change −1.31 and 6PPD-Q fold change 1.95) were both dysregulated (Supplemental Table S3). The *GSY* family encodes glycogen synthases, which contribute heavily to overall glycogen production [49]. The *GSY1* promoter has also been reported as being complex, which could offer an advantage to cellular response under environmental stressors [50]. Ndi1, a subunit of complex I, has previously been linked to preventing the generation of reactive oxygen species (ROS), a common stress response radical [51,52,53]. When exposed to 6PPD, *NDI1* was downregulated (fold change −1.27) but upregulated in response to 6PPD-Q exposure (fold change +1.30) (Supplemental Table S3). Under environmental stress conditions, like the addition of a xenobiotic substance, dsDNA breaks are more prevalent [54]. The gene *RAD59* is responsible for double-strand break DNA repair [55]. The addition of 6PPD induced a −1.97-fold change, and 6PPD-Q induced a +1.75-fold change. This further supports the notion of differing mechanisms of action between the two PPDs.

### 2.4. KEGG Pathways

#### 2.4.1. 6PPD and 6PPD-Q Downregulate Oxidative Phosphorylation KEGG Pathway Genes

**Figure 8:**
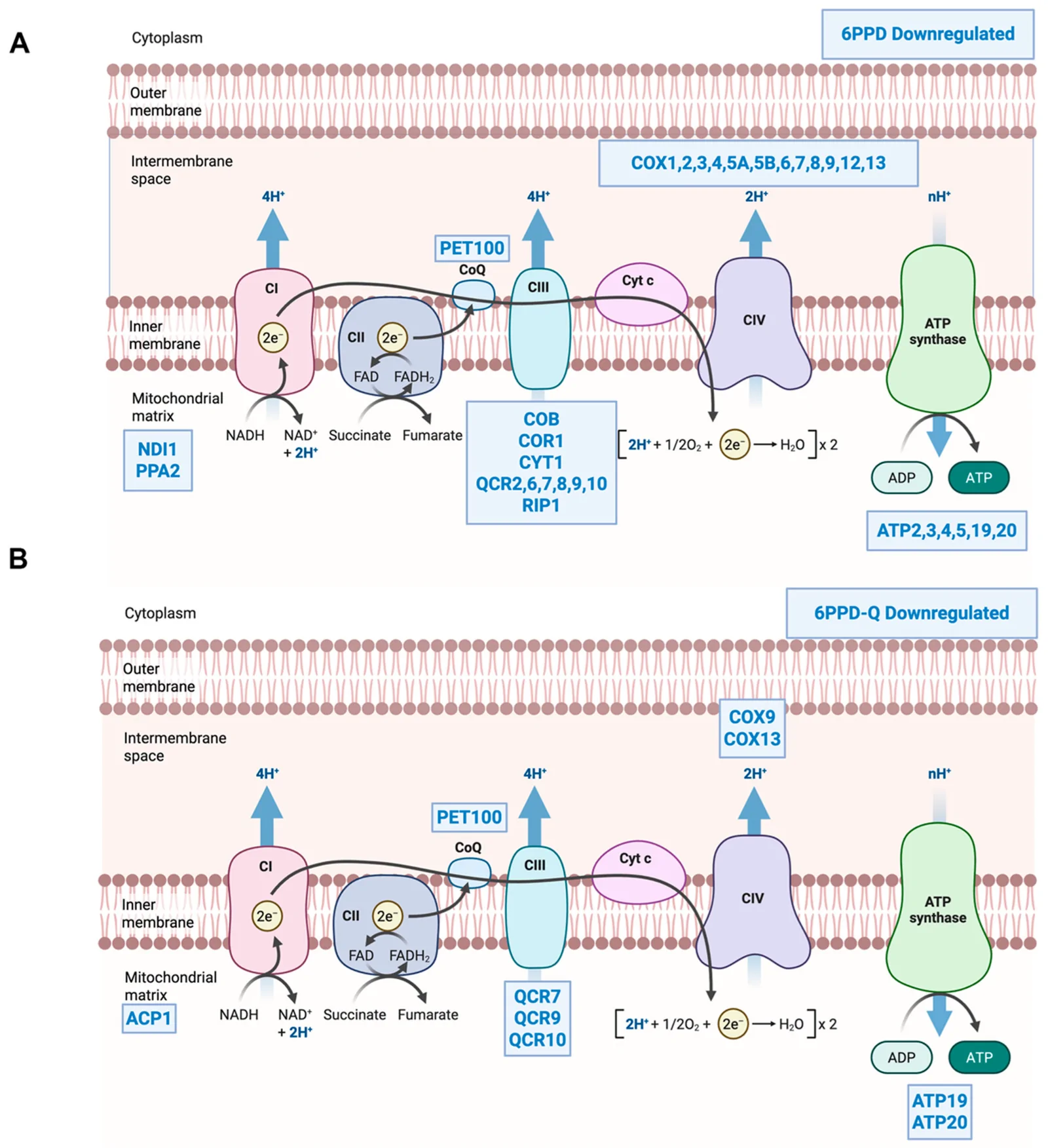
Downregulated genes associated with the oxidative phosphorylation pathway. (**A**) Downregulated genes (33) associated with the mitochondrial matrix, Complex III (CIII), Complex IV (CIV), and ATP synthase (Complex V) after exposure to 6PPD. (**B**) Downregulated genes (8) associated with the mitochondrial matrix, Complex III (CIII), Complex IV (CIV), and ATP synthase (Complex V) after exposure to 6PPD-Q. Arrows represent the movement of electrons, protons, and related molecules through the electron transport chain. Downregulated genes are shown in blue. Created using Biorender.com.

After exposure to 6PPD and 6PPD-Q, there was a distinct trend in the downregulation of genes associated with the oxidative phosphorylation pathway. A total of 31 genes were downregulated upon exposure to 56 µM 6PPD (Figure 8A). The downregulated genes fell into the following categories: 2 genes associated with the mitochondrial matrix (*NDI1*, *PPA2*), 1 gene for Coenzyme Q (*PET100*), 10 genes for Complex III (*COB*, *COR1*, *CYT1*, *QCR2*, *QCR6*, *QCR7*, *QCR8*, *QCR9*, *QCR10*, *RIP1*), 12 for Complex IV (*COX1*, *COX2*, *COX3*, *COX4*, *COX5A*, *COX5B*, *COX6*, *COX7*, *COX8*, *COX9*, *COX12*, *COX13*), and 6 genes associated with the ATP synthase or Complex V (*ATP2*, *ATP3*, *ATP4*, *ATP5*, *ATP19*, *ATP20*). The functions and fold changes for each gene have been listed in Table 2.

An amount of 50 µM of 6PPD-Q also impacted genes related to the oxidative phosphorylation pathway; however, fewer genes were affected compared to 6PPD (Figure 8B). There was a total of nine genes downregulated, with one gene being associated with the mitochondrial matrix (*ACP1*), one gene for Coenzyme Q (*PET100*), three genes for Complex III (*QCR7*, *QCR9*, *QCR10*), two genes for Complex IV (*COX9*, *COX13*), and two genes for the ATP synthase (*ATP19*, *ATP20*). The functions and fold changes for each gene are listed in Table 3.

#### 2.4.2. 6PPD Upregulates Arginine Biosynthesis KEGG Pathway Genes

**Figure 9:**
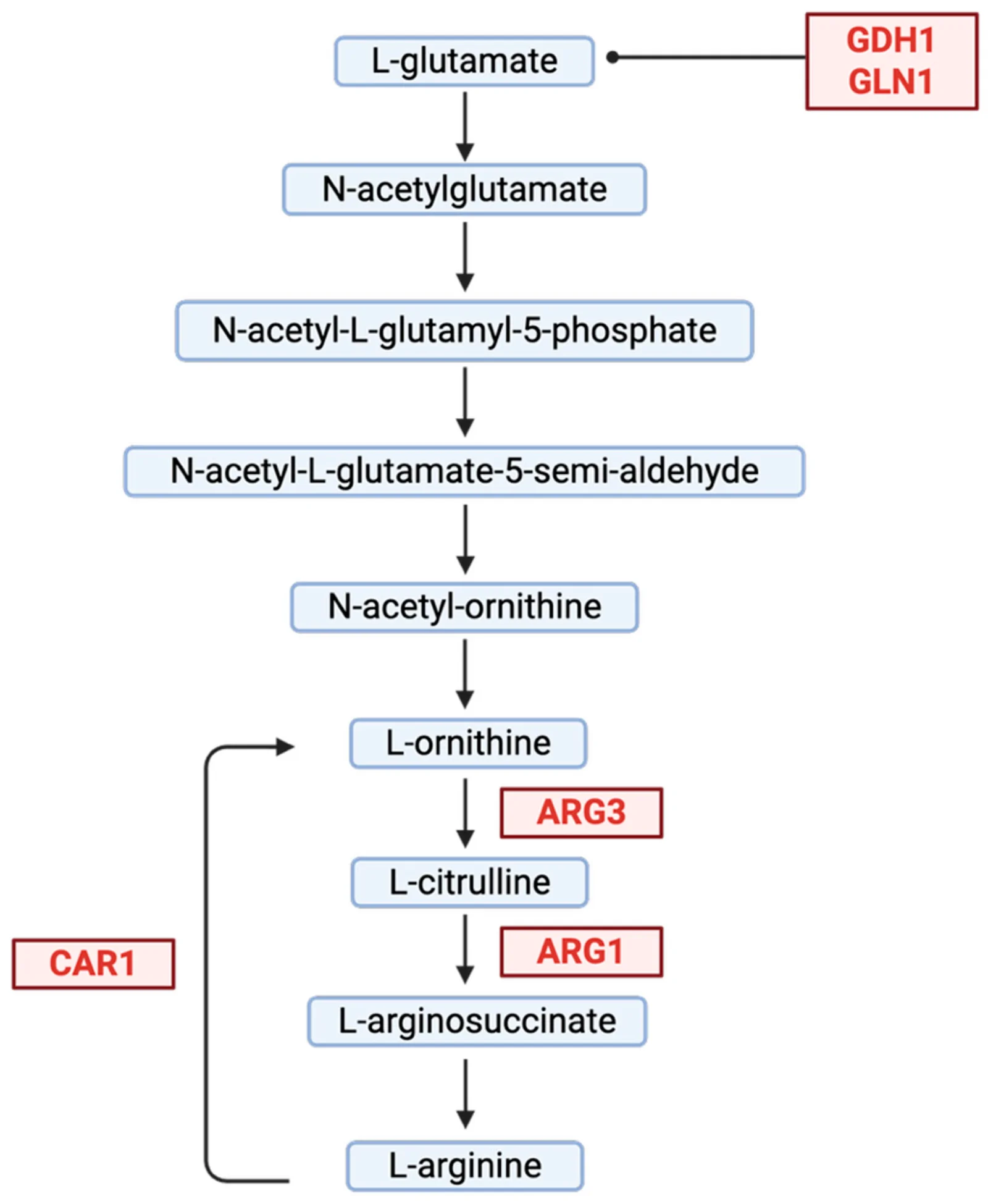
Diagram of the yeast arginine biosynthesis pathway. 6PPD upregulated genes are shown in red. Created using Biorender.com.

When analyzing the upregulated genes for 6PPD, there were five genes associated with the arginine biosynthesis pathway (Figure 9). The first step in arginine biosynthesis involves L-glutamate transforming into N-acetylglutamate, which had two upregulated genes (*GDH1*, *GLN1*). Step five, L-ornithine to L-citrulline, and step six, L-citrulline to L-arginosuccinate, had one upregulated gene each (*ARG3*, *ARG1*), respectively. *CAR1*, a gene responsible for a feedback loop converting L-arginine back into L-ornithine, was also upregulated. Key functions and fold changes for each gene are listed in Table 4.

It is worth noting that two arginine biosynthesis genes, *ARG1* and *ARG3*, were not only implicated as being upregulated by 6PPD but also downregulated by 6PPD-Q (Supplemental Table S4). Arginine and intermediates protect yeast during stress conditions by acting as ROS scavengers, nitric oxide precursors, and polyamide precursors, making the dysregulation of those two genes an interesting discovery [56,57,58].

#### 2.4.3. 6PPD-Q Upregulates Cystine and Methionine Biosynthesis KEGG Pathway Genes

**Figure 10:**
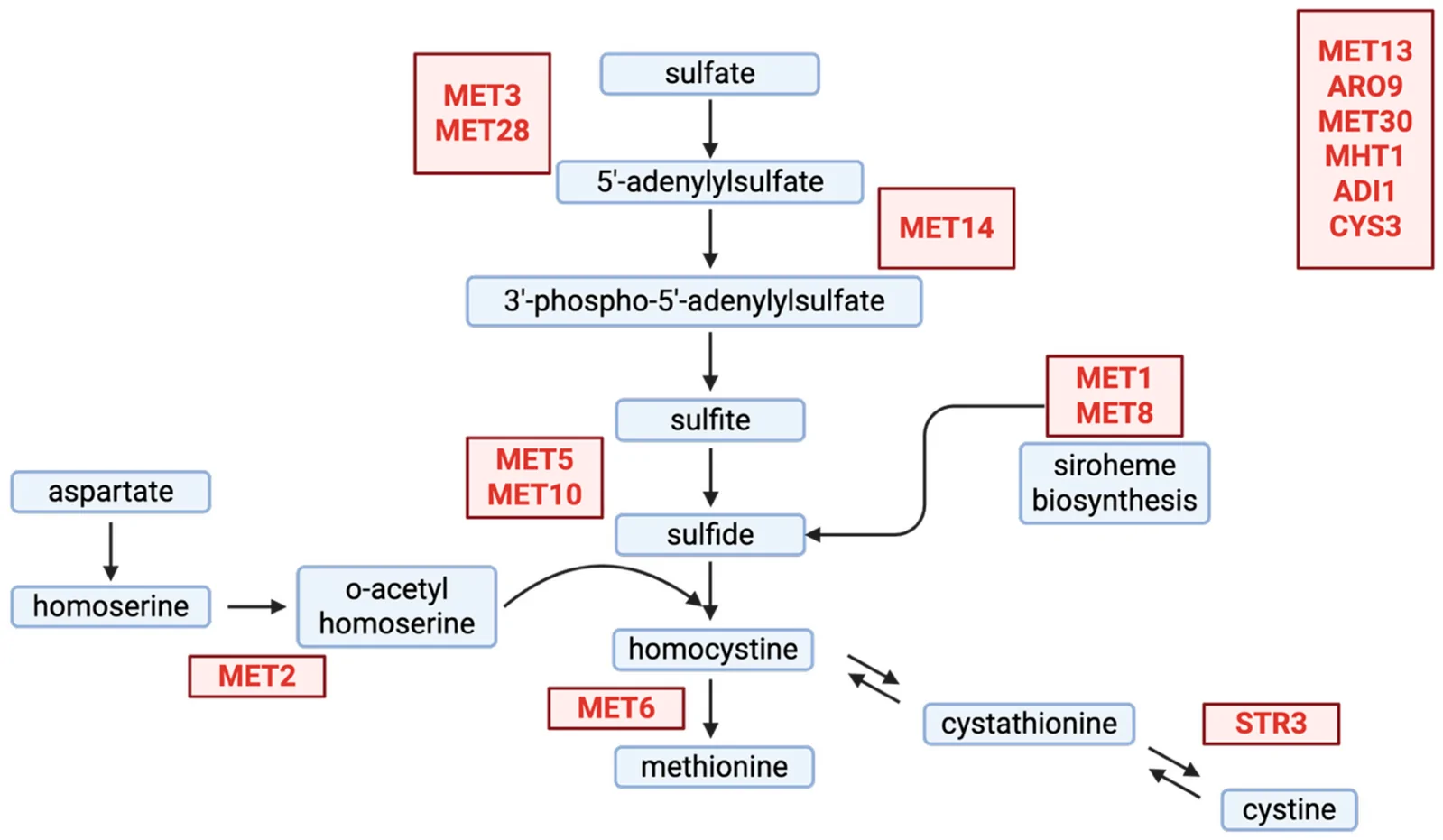
Diagram of yeast cysteine and methionine biosynthesis pathway. 6PPD-Q upregulated genes are shown in red. Additional upregulated genes associated with the pathway are shown in the box. Arrows represent movement of intermediates through the biosynthesis pathway. Created using Biorender.com.

Like 6PPD, 6PPD-Q also showed trends of upregulating KEGG pathways, specifically the cystine and methionine biosynthesis pathway. In total, there were 10 genes directly involved in the pathway (*MET1*, *MET2*, *MET3*, *MET5*, *MET6*, *MET8*, *MET10*, *MET14*, *MET28*, and *STR3*) and 6 genes associated with the pathway (*ADI1*, *ARO9*, *CYS3*, *MET13*, *MET30*, and *MHT1*) (Figure 10). The functions and fold changes for each gene are listed in Table 5. Like the arginine biosynthesis pathway, the cysteine and methionine biosynthesis pathway also has implications in cellular stress response [59].

### 2.5. Mitochondrial Staining

**Figure 11:**
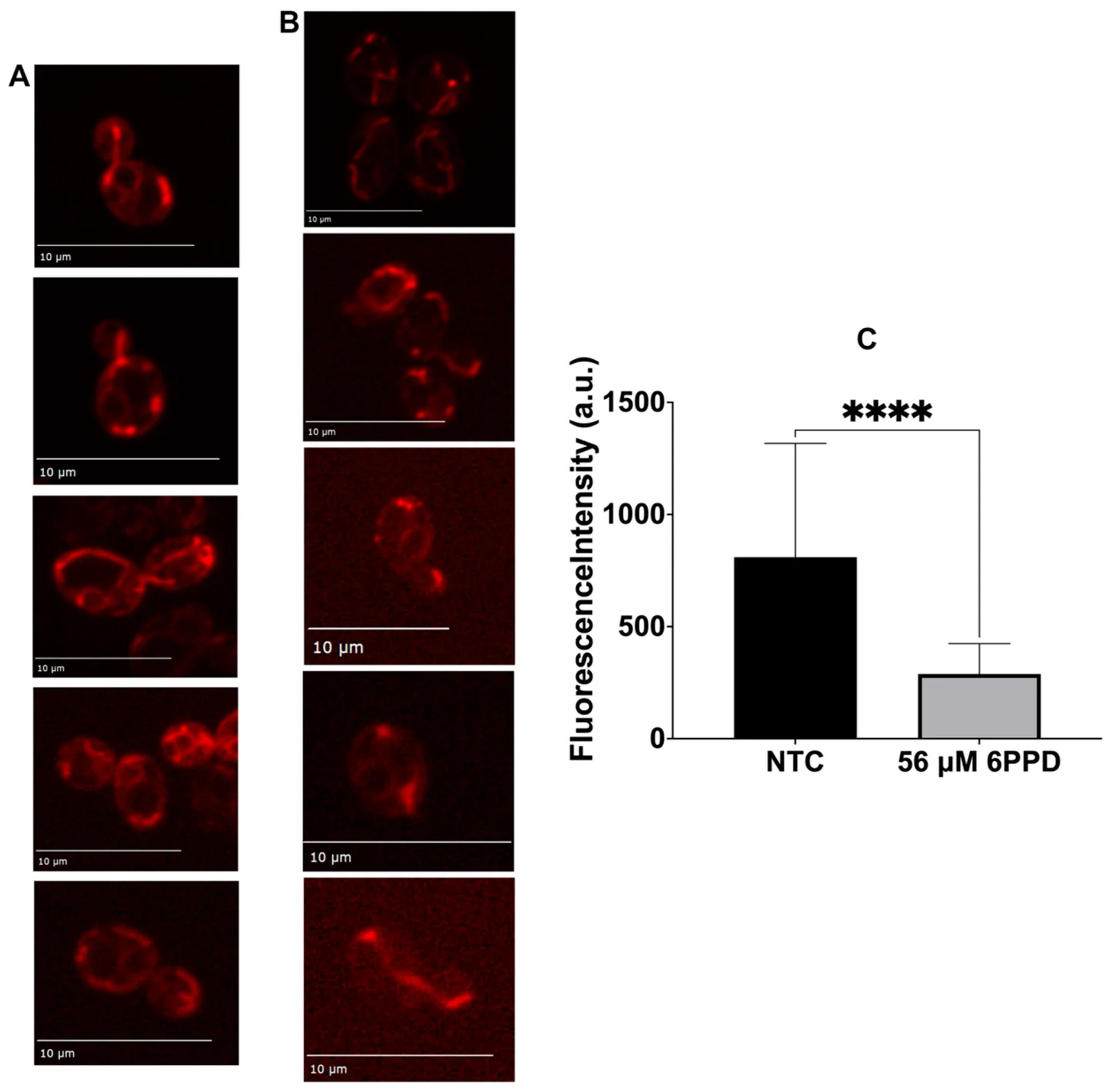
Mitochondrial staining to determine net fluorescent intensity. (**A**) Non-treated control representative images. (**B**) An amount of 56 µM treated representative images. (**C**) Mitochondria fluorescence intensity after 6-h exposure to IC_50_ (56 µM) 6PPD, then treatment with 250 nM Mito Tracker Red. Scale bar (**A**,**B**) is 10 µm. **** *p* < 0.0001, *n* = 27.

To further investigate the decrease in growth in the presence of 6PPD, the mitochondria were stained for analysis. Samples were treated with 56 µM 6PPD for 6 h before staining with Mito Tracker Red. Both PPDs have been shown to affect oxidative stress, metabolism, and the P450 metabolic enzymes in other model organisms [41,60,61]. When exposed to 56 µM of 6PPD, the mitochondrial fluorescent intensity was significantly lower than that of the non-treated samples (Figure 11A). Mito Tracker Red fluorescence intensity is directly correlated to the membrane potential created by the electron transport chain energy production pathway. The dye accumulates in the mitochondrial matrix, indicating that, as the metabolic rate increases, so does the accumulation of dye. The decrease in mitochondrial fluorescent intensity after treatment with 6PPD is likely due to decreased metabolism and energy production, which correlates with the decreased growth previously reported (Figure 11B,C). Additional mitochondrial staining was performed with 50 µM 6PPD-Q; however, results were unsuccessful. It is possible the aforementioned agglomeration of 6PPD-Q in YPD could have impacted the ability of Mito Tracker Red to enter the yeast cells.

### 2.6. RT-qPCR Analysis

**Figure 12:**
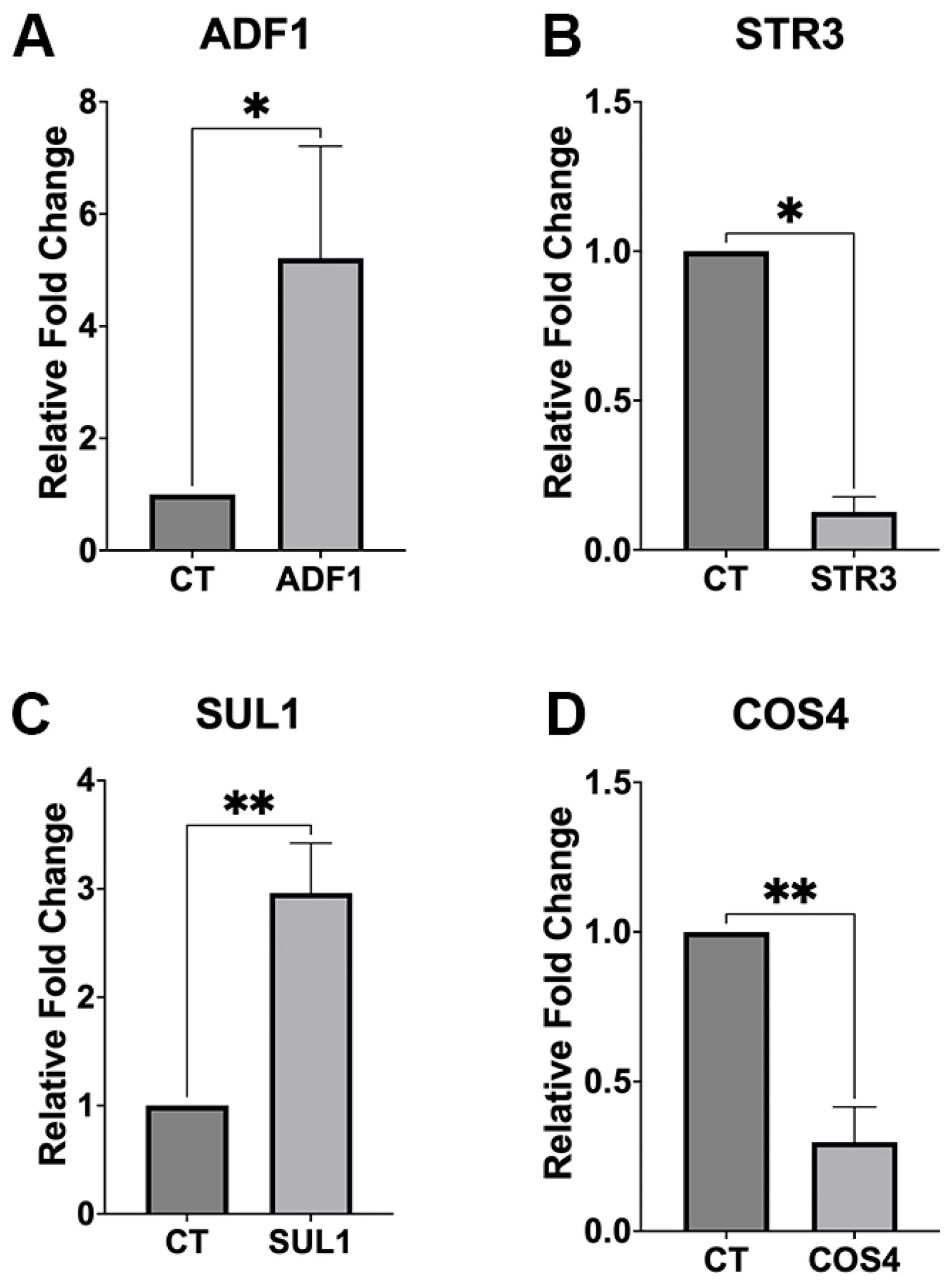
Verification of RNA sequencing using RT-qPCR. RT-qPCR was used to determine the differential expression of transcription repressor (*ADF1*) and Cystathionine β-lyase (*STR3*) upon exposure to 56 µM 6PPD and the differential expression of high-affinity sulfate permease (*SUL1*) and endosomal protein (*COS4*) upon exposure to 50 µM 6PPD-Q as compared to the housekeeping gene *ACT1*. (**A**) Relative fold change of *ADF1*. (**B**) Relative fold change of *STR3*. (**C**) Relative fold change of *SUL1*. (**D**) Relative fold change of *COS4*. Data are presented as means ± SD, * *p* < 0.05, ** *p* < 0.01, and *n* = 3.

Using RT-qPCR, the reproducibility of the RNA-seq data was evaluated. The non-differentially expressed gene *ACT1*, actin, is a structural component of the cytoskeleton, and was used as a reference. For 6PPD, two DEGs were chosen, *ADF1* and *STR3*. Adf1 is a transcription repressor with implications in yeast mating [62]. The relative fold change for *ADF1* increased to 5.211 ± 1.162 compared to the control (Figure 12A). *STR3* encodes a cystathionine β-lyase, which has a prominent role in sulfur amino acid metabolism [63]. The relative fold change for *STR3* was significantly reduced to 0.1277 ± 0.2113 compared to the control (Figure 12B).

For 6PPD-Q, two DEGs were chosen, *SUL1* and *COS4*. *SUL1* encodes a high-affinity sulfate transporter that has implications in regulating lifespan [64,65]. There was a significant upregulation in *SUL1* expression with a relative fold change of 2.961 ± 0.2938 (Figure 12C). As for *COS4*, this gene is believed to be an endosomal protein involved in multivesicular sorting [66,67,68]. The expression of *COS4* was low between the control and treated sample, but there was still a significant decrease in expression shown by the relative fold change of 0.2980 ± 0.1045 (Figure 12D).

## 3. Discussion

As more information about 6PPD and 6PPD-Q toxicity is published, there is mounting concern for environmental and human health. Because yeast has prominent roles in the environment and human health, it offers a unique avenue to discover toxicological information about PPDs. Thus far, this is the first study to investigate the toxic effects of 6PPD and 6PPD-Q in *Saccharomyces cerevisiae* and to reveal possible agglomeration of PPDs. Our data revealed the gene expression changes in both PPDs, and the influence on the yeast stress response and the oxidative phosphorylation pathway, likely contributing to hindered yeast proliferation. Additionally, 6PPD was revealed to be more toxic than 6PPD-Q regarding yeast proliferation and overall gene dysregulation. This discrepancy between the two PPDs could be attributed to the tendency of 6PPD-Q to agglomerate in YPD media. Future studies investigating cellular uptake and metabolism of PPDs could corroborate this hypothesis. This study aimed to compare 6PPD and its transformation product 6PPD-Q in the hope of gaining insight into their molecular mechanism and to support future environmental regulations.

### 3.1. 6PPD and 6PPD-Q Agglomeration in Media

The solubility of 6PPD-Q greatly impacts its stability and toxicity. 6PPD-Q has relatively poor solubility but can remain stable in simple aqueous solutions [69,70]. Given its poor solubility but high stability, this allows for 6PPD-Q to travel well in the environment [5]. To the best of our knowledge, there are few studies reporting on the agglomeration of 6PPD and 6PPD-Q in different media, which is why we chose to investigate both PPDs in SD and YPD media. The solubility of both PPDs relies greatly on the medium and pH. Upon the discovery of 6PPD-Q agglomeration and heightened background absorbance, we took this into account when calculating the minimal inhibitory concentration. Since pH and media are two factors that change greatly depending on model organism or cell line, we speculate that reported instances of increased growth after exposure to 6PPD, or 6PPD-Q could be attributed to agglomeration. Several studies have reported that both PPDs have promoted cellular growth in human hepatocellular carcinoma (HepG2), non-small cell lung cancer (NSCLC), and colorectal cancer (Huh7) cell lines [37,39,71]. Depending on media and pH, the increase in cell proliferation could be attributed to 6PPD or 6PPD-Q background absorbance, meaning the reported inhibitory concentrations are underestimates. In this study, only the background absorbance for 6PPD-Q was removed because 6PPD did not greatly increase cellular growth or background absorbance. Additionally, it is important to note that the working concentrations we used are much higher than environmentally relevant concentrations, which could contribute to PPD agglomeration.

### 3.2. 6PPD and 6PPD-Q Induce Yeast Stress Responses

In yeast, the largest amount of energy production stems from the oxidative phosphorylation pathway, or aerobic respiration, in the mitochondria [72,73]. Without the electron transport chain, yeast must rely on fermentation, greatly reducing its ATP production capacity. Through RNA sequencing, we identified the oxidative phosphorylation pathway to be dysregulated in the presence of 6PPD and 6PPD-Q. After 6PPD exposure, there was a significant decrease in mitochondrial fluorescence, which is positively correlated with energy production (Figure 11). Our findings align with studies reporting decreased mitochondrial function in *C. elegans*, an aquatic and terrestrial model organism, suggesting a similar mechanism of action of 6PPD in yeast and nematodes [32]. In yeast, Complexes III, IV, and V had the most dysregulated genes. Similarly, in *C. elegans*, 6PPD-Q was found to inhibit Complex I, II, III, IV, and V activity [74,75,76,77]. In yeast, we found that the *COX* gene family, associated with Complex IV, was significantly downregulated by 6PPD (Figure 8A, Table 1) while only two *COX* genes were downregulated by 6PPD-Q (Figure 8B, Table 2). Between organisms, the *COX* genes are highly conserved, making comparisons easy [78,79]. In *C. elegans*, the gene *cox-4*, an analogue to the *COX-4* gene in yeast, had increased expression upon exposure to 6PPD-Q [76]. Between nematodes and yeast, there is a similar trend in the oxidative phosphorylation pathway being dysregulated by PPDs. This suggests there may be some similarities in the mechanism of action between 6PPD and 6PPD-Q. However, without the addition of mitochondrial staining after exposure to 6PPD-Q it is difficult to draw a conclusion on any specific mechanism of action. It is worthwhile mentioning that, in our study, we used the minimal inhibitory concentration of 50 µM 6PPD-Q, which could explain why fewer genes associated with the oxidative phosphorylation pathway are affected. While our focus has been on the oxidative phosphorylation pathway, it is important to point out that among the dysregulated genes there were other stress response pathways implicated. 6PPD and 6PPD-Q have both up- and downregulated genes associated with the primary stress response (*MPC3*, Figure 7), DNA repair and replication stress (*RAD59*, Supplemental Table S3), oxidative stress resistance (*QCR7*, Figure 7 and Figure 8), osmotic stress (*HXT11*), general metabolic stress and nutrient starvation (*ARG3*, Figure 9, *MET1*, Figure 10) and fatty acid accumulation (*ERG3*, Figure 7). While this does support the widespread toxic effects of the PPDs in yeast, among these other stress response pathways only a few genes were implicated in each.

### 3.3. Metabolic Fate of 6PPD and 6PPD-Q

The metabolic fate of 6PPD and 6PPD-Q remains unclear. Originally, 6PPD was thought to only transform into 6PPD-Q in the presence of ozone. This has since been disproved as 6PPD-Q was detected as an in vitro transformation product of 6PPD [60,80]. These findings highlight that exposure to just 6PPD could also mean exposure to 6PPD-Q. Given this discovery, it is possible that yeast cells can metabolize 6PPD into 6PPD-Q or an equally toxic transformation product, explaining the high toxicity of 6PPD, comparatively. It is also possible that the acute toxicity seen with 6PPD-Q at the minimal inhibitory concentration of 50 µM could be from non-agglomerated 6PPD-Q entering the yeast and causing metabolic decline. Additionally, the reported overall net negative charge of −13.2 mV of 6PPD-Q in YPD media (Table 1) could also be a contributing factor to the lower toxicity. As yeast have an overall negative surface charge, it is possible that 6PPD-Q experiences strong electrostatic propulsion. Until mass spectroscopy metabolomics are performed in yeast, the internalization remains unclear. It has been reported several times that 6PPD and 6PPD-Q can interact with cytochrome P450, which could be responsible for at least one step of metabolism [80,81,82,83,84]. While there are analogous cytochrome P450 genes in yeast, they are limited in number and mainly associated with the ergosterol biosynthesis pathway [85]. We saw decline in energy production after exposure to both PPDs, but this is not the only negative impact on metabolism reported in the literature. Another prominent target for 6PPD and 6PPD-Q is the dysregulation of lipid metabolism, specifically in mice, black-spotted frogs, nematodes, zebrafish, and hepatocytes [15,86,87,88,89,90]. This supports the notion that there are species-specific and different metabolic fates for both PPDs.

## 4. Materials and Methods

### 4.1. Yeast Strain and Growth Conditions

The wild type yeast strain S288c (*Saccharomyces cerevisiae*) used for this study was previously prepared to express actin-binding protein 1 tagged with green fluorescent protein (Abp1-GFP) [91]. To prepare fresh cultures, yeast taken from storage at −80 °C was streaked on yeast peptone dextrose (YPD) agar plates. The plate was incubated at 30 °C. An isolated colony was selected and transferred to the corresponding liquid media. The liquid culture was then incubated overnight or for up to 24 h in a temperature-controlled shaker at 30 °C and 250 revolutions per minute (rpm). The fresh overnight stock was diluted to an optical density (OD) of 0.1 and used for subsequent experiments.

### 4.2. Chemical Preparation

The chemicals 6PPD (Cayman, Ann Arbor, MI, USA, Cat. 38246) and 6PPD-Q (Cayman, Ann Arbor, MI, USA Cat. 38247) were sourced from Cayman Chemical (Ann Arbor, MI, USA). Both were reconstituted in pure DMSO. 6PPD had a final stock concentration of 3 mg/mL (11.0 mM). 6PPD-Q had a final stock concentration of 5 mg/mL (16.73 mM).

### 4.3. Growth Assay

Samples of Abp1-GFP cells were grown in YPD media for 6 h after treatment with 6PPD (200 µM, 100 µM, 50 µM, 25 µM, and 12.5 µM). An additional growth assay with treatment groups containing the 1.8% DMSO in all 6PPD concentrations was completed. For treatment of 6PPD-Q (100 µM, 50 µM, 25 µM, 12.5 µM, and 6.25 µM), 1 mL cultures of Abp1-GFP were used. Samples containing 20, 1.8, and 0.6% DMSO were used as controls. Following incubation, the sample’s optical density was recorded at 600 nm. Viability data were used to calculate the IC_50_ value for 6PPD.

### 4.4. Kinetic Growth Assay

A fresh stock of Abp1-GFP cells was grown in YPD overnight and diluted to 0.01 optical density. A total of 100 µL of yeast stock was added to a round-bottom 96-well plate (Corning Incorporated, Kennebunk, ME, USA). A dilution gradient of 6PPD was added in quintuplicate (200 µM, 100 µM, 50 µM, 25 µM, and 12.5 µM). Measurements were recorded at 594 nm, every 30 min, over a 24-h period using the BioTEK ELx808 Incubating Absorbance Plate Reader (BioTek, Winooski, VT, USA) equipped with Gen5 Microplate Reader and Imager Software 3.03 (BioTek, Winooski, VT, USA). Data was graphed using Prism GraphPad 9.0.

### 4.5. 6PPD and 6PPD-Q Agglomeration

#### 4.5.1. Dynamic Light Scattering and Zeta Potential

6PPD and 6PPD-Q agglomeration was tested using deionized water (pH 5, 6, 7, and 8), sodium phosphate (dibasic) buffer (pH 5, 6, 7, and 8), synthetic defined (SD) media, and YPD media. Several 1 mL samples of 56 µM 6PPD and 50 µM 6PPD-Q were created in each medium. Dynamic light scattering was performed to analyze the size of the particle agglomeration using a Zetasizer Nano zs90 (Malvern, Worcestershire, UK). The estimated refractive index (RI) for 6PPD is 1.6300 (www.zhonganindustry.com, accessed 28 August 2026). This RI was used for both chemicals. Each sample was measured 10 times in triplicate. To determine the surface charge, the same instrumentation was used with Malvern Zetasizer Nano Series disposable folded capillary cells (Malvern, Worcestershire, UK, Cat. DTS1070). The IC_50_ (56 µM) and minimal inhibitory concentration (50 µM) for 6PPD and 6PPD-Q, respectively, were measured in YPD. Each sample was run in triplicate.

#### 4.5.2. Fluorescence Assay

Intrinsic fluorescence of 6PPD and 6PPD-Q was tested using SD and YPD media. Final concentrations in each medium were 56 µM 6PPD and 50 µM 6PPD-Q. Emission was scanned from 350 to 700 nm using a PTI spectrofluorometer (PTI Photon Technology International, Birmingham, NJ, USA) with excitation wavelength 280 nm. The absorbance of both 6PPD (200, 100, 50, 25, 12.5 µM) and 6PPD-Q (100, 50, 25, 12.5, 6.25 µM) was also tested after 6 h of incubation at 30 °C. Measurements were taken in triplicate using a 96-well plate reader (BioTek Instruments, Winooski, VT, USA) at 450–630 nm.

### 4.6. RNA Sequencing

Quadruplicate yeast cultures of 2 mL with an optical density of 0.1 were created and treated with 56 µM 6PPD and 50 µM 6PPD-Q for 4.5 h. After incubation, RNA was extracted following the MasterPure Yeast RNA Purification Kit (Lucigen, Middleton, WI, USA, Cat. MPY03100) protocol. The RNA concentration and quality were checked with an Implent Nanophotometer P330 (Westlake Village, CA, USA) before being sent to Novogene Lab in California for next-generation sequencing. The FASTQ raw data files were uploaded and concatenated using Galaxy (usegalaxy.org, accessed on 25 August 2026). Next, the concatenated files were uploaded to the QIAGEN CLC Genomic Workbench 23 to be checked for quality control. The reference genome of *Saccharomyces cerevisiae* was used to identify differentially expressed genes (DEGs) with a fold change of ±1.25 and *p* < 0.05. The identified genes were categorized into protein classes and cellular processes using Panther Gene Ontology (pantherdb.org, accessed 28 August 2026). Bonferroni correction was employed to rule out false-positive interpretations.

### 4.7. Primer Design

Based on up- and downregulated gene analysis using Panther Gene Ontology, genes were selected based on function and magnitude of fold change. Each primer had the following parameters: 70–200 bps, melting range of 50–55 °C, max amplicon of 200 bps, GC content between 50 and 55%, and a GC clamp of 1. The selected primers were ordered from Integrated DNA Technologies (IDT, Coralville, IA, USA).

### 4.8. Mitochondrial Staining and Confocal Imaging

Fresh Abp1-GFP culture was diluted to an OD 0.1 in 3 mL YPD. For 6PPD treatment, the IC_50_ concentration of 56 µM was used. For 6PPD-Q treatment, the minimal effective concentration (50 mM) was used for treatment. Samples were grown for 6 h at 30 °C and 250 rpm. After incubation, the samples were centrifuged and washed twice with 1 mL of fresh YPD media. Then, the pellet was resuspended in 1 mL YPD media containing 250 nM Mito Tracker Red dye (Thermo Fischer Scientific, Waltham, MA, USA Cat. M22425). The yeast and dye were incubated for 30 min, then washed with YPD media to remove excess dye. The stained samples were resuspended in 1.5 mL of fresh YPD. Fluorescence microscopy images were then taken using the Olympus IX-81 inverted microscope (Olympus Corporation, Tokyo, Japan) equipped with a Yokogawa CSU-X1 spinning confocal box (Yokogawa, Sugar Land, TX, USA) and ImagEM camera (Hamamatsu Photonics, Shizuoka, Japan). The images were taken with the 100X oil objective, the SDC single filter set, and the red laser at 561 nm. The mitochondrial fluorescence was analyzed using the line function in SlideBook 2025.

### 4.9. RT-qPCR

To start, cDNA was synthesized for each sample in quadruplicate following the SuperScript IV cDNA synthesis kit instructions (Thermo Fisher Scientific, Waltham, MA, USA, Cat. 18091050). The cDNA concentrations were then quantified using a dsDNA Qubit Assay Kit and a Qubit Fluorometer 3.0. For cDNA amplification, the Applied Biosystems QuantoStudio 5 Real-Time PCR System (Applied Biosystems, Thermo Fisher Scientific, Waltham, MA, USA) was used. A primer efficiency test was performed using the housekeeping gene *ACT1* to determine optimal cDNA concentration for amplification. After amplification, C_T_ values for each gene were assessed using QuantoStudio Design & Analysis Software 2.8.0. Faster amplification, a lower C_T_ value, indicates upregulation of the target gene compared to the control; slower amplification, a higher C_T_ value, indicates downregulation of the target gene compared to the control. C_T_ values for the housekeeping gene between the treated and control groups were used to verify. Biological replicates were tested in triplicate with duplicate technical replicates for each gene. The double delta (2^−∆∆CT^) method was used to determine the relative fold change for each gene [92].

### 4.10. Statistical Analysis

Data graphs were produced using Prism GraphPad 9.0 using the one-way ANOVA, Dunnett’s multiple comparisons *t*-test, and unpaired *t*-test. Each experiment was completed in triplicate unless otherwise noted. Variation within data sets is represented by error bars. Statistical significance is represented as * *p* < 0.05, ** *p* < 0.01, *p* *** < 0.001, and *p* **** < 0.0001 within each graph.

## 5. Conclusions

Through this work, we investigated the effects of 6PPD and 6PPD-Q on *Saccharomyces cerevisiae*. From our series of experiments, we were able to determine that 6PPD inhibits yeast proliferation and dysregulates a myriad of oxidative phosphorylation genes and additional stress response related genes. On the other hand, 6PPD-Q seemingly induced cellular growth, which was explained by the chemical’s tendency to agglomerate in YPD media. 6PPD-Q also impacted oxidative phosphorylation genes and stress response genes. Collectively, this work revealed the agglomeration of 6PPD and 6PPD-Q in growth mediums, widespread cellular stress response, and their impact on metabolic and oxidative phosphorylation pathway gene regulation in *Saccharomyces cerevisiae*.

## Figures and Tables

**Table 1:** Zetapotential for 6PPD and 6PPD-Q in YPD media.

Sample	Run 1	Run 2	Run 3	Average (mV)
YPD	−6.78	−8.45	−9.00	−8.08
YPD + 56 µM 6PPD	−7.57	−8.28	−9.13	−8.33
YPD + 50 µM 6PPD-Q	−12.5	−13.7	−13.3	−13.2

**Table 2:** 6PPD downregulated genes associated with oxidative phosphorylation.

	Gene	Function	Fold Change
Mitochondrial Matrix	NDI1	NADH: ubiquinone oxidoreductase	−1.27
	PPA2	Mitochondrial inorganic pyrophosphatase	−1.28
Coenzyme Q	PET100	Chaperone that facilitates the assembly of cytochrome c oxidase	−1.27
Complex III	COB	Cytochrome b	−1.26
	COR1	Core subunit of the ubiquinol-cytochrome c reductase complex	−1.51
	CYT1	Cytochrome c1	−1.40
	QCR2	Subunit 2 of ubiquinol cytochrome-c reductase	−1.41
	QCR6	Subunit 6 of the ubiquinol cytochrome-c reductase complex	−1.34
	QCR7	Subunit 7 of ubiquinol cytochrome-c reductase	−1.32
	QCR8	Subunit 8 of ubiquinol cytochrome-c reductase	−1.26
	QCR9	Subunit 9 of ubiquinol cytochrome-c reductase	−1.26
	QCR10	Subunit of the ubiquinol-cytochrome c oxidoreductase complex	−1.26
	RIP1	Ubiquinol-cytochrome-c reductase	−1.31
Complex IV	COX1	Subunit I of cytochrome c oxidase	−1.26
	COX2	Subunit II of cytochrome c oxidase	−1.26
	COX3	Subunit III of cytochrome c oxidase	−1.26
	COX4	Subunit IV of cytochrome c oxidase	−1.37
	COX5A	Subunit Va of cytochrome c oxidase	−1.26
	COX5B	Subunit Vb of cytochrome c oxidase	−1.62
	COX6	Subunit VI of cytochrome c oxidase	−1.26
	COX7	Subunit VII of cytochrome c oxidase	−1.27
	COX8	Subunit VIII of cytochrome c oxidase	−1.26
	COX9	Subunit VIIa of cytochrome c oxidase	−1.26
	COX12	Subunit VIb of cytochrome c oxidase	−1.26
	COX13	Subunit VIa of cytochrome c oxidase	−1.26
ATP Synthase	ATP2	Beta subunit of the F1 sector of mitochondrial F1F0 ATP synthase	−1.39
	ATP3	Gamma subunit of the F1 sector of mitochondrial F1F0 ATP synthase	−1.29
	ATP4	Subunit b of the stator stalk of mitochondrial F1F0 ATP synthase	−1.28
	ATP5	Subunit 5 of the stator stalk of mitochondrial F1F0 ATP synthase	−1.46
	ATP19	Subunit k of the mitochondrial F1F0 ATP synthase	−1.28
	ATP20	Subunit g of the mitochondrial F1F0 ATP synthase	−1.43

**Table 3:** 6PPD-Q downregulated genes associated with oxidative phosphorylation.

	Gene	Function	Fold Change
Mitochondrial Matrix	ACP1	Mitochondrial matrix acyl carrier protein	−1.42
Coenzyme Q	PET100	Chaperone that facilitates the assembly of cytochrome c oxidase	−1.41
Complex III	QCR7	Subunit 7 of ubiquinol cytochrome-c reductase	−1.30
	QCR9	Subunit 9 of ubiquinol cytochrome-c reductase	−1.26
	QCR10	Subunit of the ubiquinol-cytochrome c oxidoreductase complex	−1.51
Complex IV	COX9	Subunit VIIa of cytochrome c oxidase	−1.39
	COX13	Subunit VIa of cytochrome c oxidase	−1.25
ATP Synthase	ATP19	Subunit k of the mitochondrial F1F0 ATP synthase	−1.29
	ATP20	Subunit g of the mitochondrial F1F0 ATP synthase	−1.31

**Table 4:** 6PPD upregulated genes associated with the arginine biosynthesis pathway.

Gene	Key Function	Fold Change
ARG1	Argininosuccinate synthetase	2.01
ARG3	Ornithine carbamoyltransferase	1.31
CAR1	Arginase	1.72
GDH1	Glutamate dehydrogenase	2.27
GLN1	Glutamine synthetase	1.68

**Table 5:** 6PPD-Q upregulated genes associated with the cystine and methionine biosynthesis pathway.

Gene	Key Function	Fold Change
ADI1	Acireductone dioxygenase	1.41
ARO9	Aromatic aminotransferase	1.32
CYS3	Cystathionine gamma-lyase	1.26
MET1	S-adenosyl-L-methionine uroporphyrinogen III transmethylase	1.92
MET10	Subunit alpha of assimilatory sulfite reductase	1.85
MET13	Major isozyme of methylenetetrahydrofolate reductase	1.36
MET14	Adenylylsulfate kinase	1.66
MET2	L-homoserine-O-acetyltransferase	1.80
MET28	bZIP transcriptional activator in the Cbf1p-Met4p-Met28p complex	1.76
MET3	ATP sulfurylase	1.71
MET30	Nuclear F-box protein and subunit of SCF-Met30 Ub-ligase complex	1.33
MET5	Sulfite reductase beta subunit	1.65
MET6	Cobalamin-independent methionine synthase	1.36
MET8	Bifunctional dehydrogenase and ferrochelatase	1.64
MHT1	S-methylmethionine-homocysteine methyltransferase	1.78
STR3	Peroxisomal cystathionine beta-lyase	1.73

## Data Availability

Data available upon request.

## Supplementary Materials

The following supporting information can be downloaded at: https://www.mdpi.com/article/10.3390/ijms27177739/s1.

## Abbreviations

The following abbreviations are used in this manuscript:

6PPD	N-(1,3-dimethylbutyl)-N′-phenyl-p-phenylenediamine
6PPD-Q	6PPD-Quinone
PPD	p-phenylenediamine
RT-qPCR	Reverse transcriptase quantitative polymerase chain reaction
TP	Transformation product
TWP	Tire-wear particles
PM	Particulate matter
URMS	Urban runoff mortality syndrome
7PPD	N-(1,4-dimethylpentyl)-N′-phenyl-p-phenylenediamine
7PPD-Q	7PPD-Quinone
77PD	N,N′-Bis(1,4-dimethylpentyl)-p-phenylenediamine
77PD	77PD-Quinone
IPPD	N-Isopropyl-N′-phenyl-p-phenylenediamine
CCPD	N,N′-dicyclohexyl-p-phenylenediamine
PPDTZ	Durazone
TMQ	Poly(1,2-dihydro-2,2,4-trimethylquinoline)
KGN	Human ovarian granulosa cell line
NSCLC	Non-small cell lung cancer cell line
L02	Human proto-hepatocyte cell line
HepG2	Hepatocellular carcinoma cell line
DMSO	Dimethyl sulfoxide
YPD	Yeast peptone dextrose
SD	Synthetic defined
DLS	Dynamic light scattering
FDR	False discovery rate
DEG	Differentially expressed genes
Huh7	Colorectal cancer cell line
