# Supplementary material for: 6PPD and 6PPD-Q Induce Mitochondrial Dysfunction in Saccharomyces cerevisiae
Source: Int J Mol Sci. 2026 Aug 29;27(17):7739. doi: 10.3390/ijms27177739 (PMC13566889; doi:10.3390/ijms27177739)
Supplement: Supplementary file 1 [file ijms-27-07739-s001.zip › ijms-4471458-supplementary.pdf]

**Supplemental Table S1.** Overlapping DEGs, 6PPD and 6PPD-Q downregulated, with associated functions and organelles. Fold changes are < -1.25 for downregulated genes. Based on DAVID Bioinformatics Functional Annotation Clustering with enrichment score > 2.0, the mitochondria and oxidative phosphorylation are the main categories downregulated by 6PPD and 6PPD-Q.

| | | | 6PPD | 6PPD-Q |
|---|---|---|---|---|
| **Gene** | **Key Functions** | **Associated Organelle** | **Fold Change** | |
| **ACS1** | Cytosolic acetyl-CoA ligase | Mitochondria | -1.36 | -1.28 |
| **ANB1** | Ribosome binding protein | Ribosome | -1.82 | -1.63 |
| **APQ12** | Integral membrane protein | Nuclear envelope, endoplasmic reticulum | -1.28 | -1.53 |
| **ATP19** | Subunit of F0 coupling factor of mitochondrial F1F0 ATP synthase | Mitochondrial inner membrane | -1.28 | -1.29 |
| **ATP20** | Subunit of F0 coupling factor of mitochondrial F1F0 ATP synthase | Mitochondrial inner membrane | -1.44 | -1.31 |
| **BUD30** | Ascospore formation and transcription activation | UNKNOWN | -1.27 | -1.50 |
| **CDD1** | Protein with cytidine deaminase activity | Nucleus, cytoplasm | -1.35 | -1.28 |
| **CGR1** | rRNA processing and ribosome biogenesis | Nucleolus | -1.39 | -1.74 |
| **CMC2** | Respiratory chain complex assembly | Mitochondrial intermembrane space, nucleus, cytoplasm | -1.37 | -1.60 |
| **COX13** | Subunit of the mitochondrial respiratory chain complex IV | Mitochondrial inner membrane | -1.26 | -1.25 |
| **COX9** | Subunit of complex IV of the mitochondrial respiratory chain | Mitochondrial inner membrane | -1.26 | -1.39 |
| **CWP2** | Structural constituent of the cell wall | Cell wall | -1.37 | -1.49 |
| **DBP2** | ATP-dependent RNA helicase | Nucleus | -1.63 | -1.37 |
| **DOC1** | Enzyme regulator of anaphase-promoting ubiquitin protein ligase complex | Mitochondria | -1.44 | -1.37 |
| **EFM1** | S-adenosylmethionine-dependent lysine methyltransferase | Cytoplasm | -1.42 | -1.28 |
| **FMP46** | UNKNOWN | UNKNOWN | -1.30 | -1.52 |
| **FMP48** | Protein kinase | Mitochondria | -1.32 | -1.37 |
| **IZH2** | A protein that negatively regulates transcription from RNA polymerase II promoter | Plasma membrane | -2.13 | -1.32 |
| **MAE1** | Mitochondrial malic enzyme | Mitochondria | -1.40 | -1.39 |

| | | | | |
|---|---|---|---|---|
| **MED11** | Subunit of the RNA polymerase II mediator complex | Nucleus | -1.27 | -1.29 |
| **MRP2** | Component of the small subunit of the mitochondrial ribosome | Mitochondrial ribosome | -1.28 | -1.27 |
| **MRPS16** | Component of the small subunit of the mitochondrial ribosome | Mitochondrial ribosome | -1.35 | -1.30 |
| **MRX14** | Putative component of the large subunit of the mitochondrial ribosome | Mitochondrial ribosome | -1.38 | -1.34 |
| **NBL1** | Subunit of the chromosomal passenger complex | Nucleus | -1.67 | -1.60 |
| **NOP9** | Nucleolar RNA-binding protein of the 90S preribosome and the ribosomal small subunit precursor preribosome | Nucleus | -1.27 | -1.27 |
| **PET100** | Protein carrier | Mitochondrial inner membrane | -1.27 | -1.41 |
| **POM33** | Integral nuclear envelope protein | Nuclear envelope | -1.25 | -1.26 |
| **PXR1** | Telomerase inhibitor | Nucleoplasm, nucleolus | -1.54 | -1.59 |
| **QCR10** | Subunit of mitochondrial respiratory chain complex III | Mitochondrial inner membrane | -1.43 | -1.51 |
| **QCR7** | Subunit of mitochondrial respiratory chain complex III | Mitochondrial inner membrane | -1.32 | -1.30 |
| **QCR9** | Subunit of mitochondrial respiratory chain complex III | Mitochondrial inner membrane | -1.26 | -1.26 |
| **RPC34** | Subunit of DNA-directed RNA polymerase III complex | Mitochondria, nucleus, cytosol | -1.30 | -1.28 |
| **RPE1** | Cytosolic ribulose-phosphate 3-epimerase | Cytosol | -1.29 | -1.25 |
| **SWC7** | Component of the nuclear Swr1 complex | Nucleus | -2.00 | -1.83 |
| **TDH2** | Glyceraldehyde-3-phosphate dehydrogenase | Cytoplasm, mitochondria, lipid particles, cell wall | -1.38 | -1.32 |
| **TIM10** | Mitochondrial protein transporter and chaperone | Mitochondrial inner membrane | -1.31 | -1.41 |
| **TMA23** | Nucleolar protein | Nucleus | -1.31 | -1.51 |
| **TRM7** | tRNA methyltransferase | Cytoplasm | -1.29 | -1.31 |
| **TRX3** | Mitochondrial disulfide oxidoreductase | Mitochondria | -1.47 | -1.32 |
| **UGA3** | Promoter proximal region, sequence-specific DNA binding protein | Nucleus | -1.40 | -1.38 |
| **WTM2** | Sequence-specific DNA-binding transcription factor | Nucleus | -1.30 | -1.30 |

| | | | | |
|---|---|---|---|---|
| **YAH1** | NAD(P)H oxidoreductase | Mitochondrial matrix | -1.36 | -1.27 |
| **YIM2** | UNKNOWN | UNKNOWN | -1.42 | -1.44 |
| **YVH1** | Protein tyrosine/serine/threonine phosphatase | Nucleus, cytoplasm | -1.32 | -1.33 |

**Supplemental Table S2.** Overlapping DEGs, 6PPD and 6PPD-Q upregulated, with associated functions and organelles. Fold changes are > 1.25 for upregulated genes. Based on DAVID Bioinformatics Functional Annotation Clustering with enrichment score > 2.0, transcription regulations via zinc ion binding and carbohydrate transport are the main categories upregulated by 6PPD and 6PPD-Q.

| | | | **6PPD** | **6PPD-Q** |
|---|---|---|---|---|
| **Gene** | **Key Functions** | **Associated Organelle** | **Fold Change** | |
| **ABF1** | DNA-binding RNA polymerase II transcription factor | Nucleus | 1.89 | 1.38 |
| **ADF1** | DNA-binding transcription repressor | Nucleus | 624 | 1541 |
| **AFR1** | Cell morphogenesis | Mating projection base, peroxisome | 1.57 | 1.66 |
| **AFT1** | DNA binding transcription factor | Cytoplasm, nucleus, kinetochores | 1.64 | 1.26 |
| **AGP1** | Transmembrane transporter | Plasma membrane | 3.57 | 1.67 |
| **APJ1** | Chaperone and ATPase activator | Cytoplasm, mitochondria, nucleus, nuclear periphery | 1.35 | 1.26 |
| **ASG7** | Mating protein | Plasma membrane | 1.63 | 1.50 |
| **ASH1** | DNA binding RNA Pol II transcription factor | Cellular bud, nucleus | 1.71 | 1.28 |
| **ATG2** | Phospholipid transfer protein | Cytoplasm, phagophore, pre-autophagosomal structure (PAS), Endoplasmic reticulum | 1.50 | 1.34 |
| **ATG7** | Atg8- and Atg12-activating enzyme | Cytosol, plasma membrane, mitochondria | 1.47 | 1.46 |
| **BCK2** | Regulation of gene expression and cell cycle | Cytoplasm, nucleus | 1.39 | 1.31 |
| **BUD28** | UNKNOWN | UNKNOWN | 1.73 | 2.17 |

| | | | | |
|---|---|---|---|---|
| **CAF120** | UNKNOWN | UNKNOWN | 2.23 | 1.45 |
| **CAT8** | Zinc cluster transcriptional activator | Cytoplasm, nucleus | 1.45 | 1.35 |
| **CHS1** | Chitin synthase | Chitosomes, plasma membrane | 2.15 | 1.28 |
| **CLN3** | Cyclin-dependent protein kinase (CDK) | Nucleus | 1.41 | 1.27 |
| **COS111** | Mitochondrial signal transduction | Mitochondria | 2.33 | 1.90 |
| **CTR2** | Transmembrane transporter | Vacuole | 1.47 | 1.29 |
| **CUP2** | DNA-binding transcription factor | Nucleus | 1.49 | 1.28 |
| **DIA1** | Invasive growth | Cytoplasm | 3.40 | 1.37 |
| **DUR1,2** | Urea and allophanate hydrolase | Cytoplasm | 1.87 | 1.26 |
| **ECL1** | UNKNOWN | UNKNOWN | 1.89 | 1.44 |
| **FET3** | Ferroxidase | Plasma membrane, endoplasmic reticulum, vacuole | 1.96 | 1.45 |
| **GAT2** | DNA binding RNA polymerase II transcription factor | UNKNOWN | 2.54 | 1.42 |
| **GIS1** | H3-K36 specific histone demethylase and sequence-specific DNA-binding transcription factor | Nucleus, cytosol | 1.38 | 1.67 |
| **GLG1** | Glycogenin glucosyltransferase | Cytosol | 1.28 | 2.51 |
| **GOR1** | Glyoxylic acid reductase | Cytoplasm, mitochondria, nucleus | 1.30 | 1.56 |
| **GUD1** | Guanine deaminase | Cytoplasm | 1.40 | 1.43 |
| **GYP7** | GTPase activator | Cytoplasm | 1.73 | 1.35 |
| **HAL5** | Protein kinase | Plasma membrane | 1.28 | 1.27 |
| **HAP4** | Transcriptional coactivator | Nucleus | 2.46 | 2.38 |
| **HBN1** | Putative oxidoreductase | Cytoplasm, nucleus | 2.71 | 1.58 |
| **HSP150** | Adenosine triphosphatase involved in cell wall organization | Cellular bud scar, cell wall, cytosol | 1.28 | 1.25 |
| **HXT1** | Hexose and pentose transmembrane transporter | Plasma membrane | 2.48 | 1.35 |
| **HXT5** | Hexose transporter | Plasma membrane | 2.06 | 1.51 |
| **HXT6** | Hexose transmembrane transporter | Mitochondria, plasma membrane | 1.56 | 2.18 |
| **ICL1** | Isocitrate lyase | Cytoplasm | 1.33 | 1.31 |
| **IMA1** | Oligo-1,6-glucosidase | Mitochondria | 1.37 | 1.51 |

| | | | | |
|---|---|---|---|---|
| **IME2** | Nuclear protein kinase | Nucleus | 1.29 | 1.49 |
| **IML2** | Clears protein aggregates | Mitochondria, cytoplasm, nucleus | 1.56 | 1.46 |
| **IRC9** | Mitotic recombination; double-strand break repair | UNKNOWN | 1.51 | 2.32 |
| **LCB3** | Dihydrosphingosine-1-phosphate phosphatase | Endoplasmic reticulum | 1.32 | 1.27 |
| **MAG1** | Damaged DNA-binding alkylbase DNA N-glycosylase | Nucleus | 1.29 | 1.42 |
| **MAL11** | Integral membrane transporter protein | Plasma membrane | 1.37 | 2.12 |
| **MAL13** | Nuclear DNA-binding transcription factor | Nucleus | 1.60 | 1.36 |
| **MAL31** | Putative alpha-glucoside transporter | Cell periphery | 1.71 | 3.04 |
| **MGA1** | Pseudohyphal growth | Nucleus | 1.56 | 2.31 |
| **MID2** | Transmembrane signaling receptor | Plasma membrane | 1.76 | 1.55 |
| **MIG1** | DNA binding transcription factor | Cytoplasm, nuclear envelope, nucleus | 1.71 | 1.83 |
| **MIG2** | RNA polymerase II proximal promoter sequence-specific DNA binding protein | Mitochondria, nucleus | 4.80 | 3.00 |
| **MIG3** | Transcriptional regulator | Chromatin | 2.02 | 1.32 |
| **MPC3** | Pyruvate transmembrane transporter | Mitochondrial membrane | 3.15 | 1.85 |
| **MTH1** | Glucose transport and signal transduction | UNKNOWN | 1.90 | 3.96 |
| **NCW2** | Wall organization and chitin localization | Cell wall, plasma membrane | 2.48 | 1.29 |
| **NGL3** | 3'-5' exonuclease | UNKNOWN | 2.40 | 1.83 |
| **NRG1** | Transcriptional repressor | Nucleus | 2.89 | 1.35 |
| **PAH1** | Mg2+-dependent phosphatidate (PA) phosphate | Vacuole, lipid particle, cytosol, nuclear membrane | 1.31 | 1.26 |
| **PCD1** | Pyrophosphatase | Peroxisome | 1.49 | 1.51 |
| **PIG1** | Regulatory subunit of the type 1 protein phosphatase | Cytoplasm | 1.39 | 1.31 |
| **PMC1** | Vacuolar Ca2+ ATPase | Vacuole | 1.54 | 1.41 |
| **POG1** | DNA-binding transcriptional activator | Nucleus | 1.44 | 1.45 |
| **PRB1** | Vacuolar proteinase B | Vacuole | 1.49 | 1.34 |
| **PRM10** | UNKNOWN | Endoplasmic reticulum | 2.00 | 1.45 |

| | | | | |
|---|---|---|---|---|
| **PRM5** | UNKNOWN | Membrane | 1.74 | 1.64 |
| **PUN1** | Plasma membrane protein | Plasma membrane, eisosome | 1.65 | 1.27 |
| **RCR1** | Plasma membrane ubiquitin ligase substrate adaptor | Plasma membrane, endoplasmic reticulum | 2.08 | 1.25 |
| **REG2** | Protein phosphatase | Cytoplasm, nucleus | 1.59 | 1.91 |
| **RHO2** | GTPase | Cell periphery | 1.36 | 1.29 |
| **RIB1** | Cyclohydrolase | Nucleus, cytoplasm | 1.51 | 1.36 |
| **RRT6** | UNKNOWN | Cytosol | 1.51 | 1.45 |
| **RTS3** | Protein serine/threonine phosphatase inhibitor | Nucleus, cytoplasm | 2.18 | 1.58 |
| **SIS2** | Subunit of the phosphopantothenoylcysteine decarboxylase complex | Nucleus | 1.30 | 1.31 |
| **SNF3** | Glucose-binding protein | Plasma membrane | 1.28 | 1.45 |
| **SRL3** | Binds specific RNA polymerase II cis-regulatory regions of DNA | Cytoplasm | 2.52 | 2.01 |
| **SSN2** | Cyclin binding protein | Nucleus | 1.26 | 1.31 |
| **STB2** | UNKNOWN | Sin3-type complex | 1.46 | 1.42 |
| **STP4** | Zinc ion binding protein | Cytoplasm, mitochondria, nucleus | 2.98 | 1.78 |
| **SUT1** | Transcription regulator | Nucleus, cytoplasm | 1.77 | 1.34 |
| **TDA1** | Protein serine/threonine kinase | Nucleus, cytoplasm | 1.27 | 1.42 |
| **TEC1** | DNA-binding RNA polymerase II transcription factor | Nucleus | 1.69 | 1.57 |
| **TIS11** | mRNA-binding translation repressor | Nucleus, cytoplasm | 3.52 | 1.82 |
| **TOS8** | Chromatin binding protein | Nucleus | 1.86 | 1.61 |
| **UBC8** | Cytoplasmic ubiquitin-protein transferase | Cytoplasm | 1.35 | 1.59 |
| **UBP9** | Putative thiol-dependent isopeptidase | Cytoplasm | 1.31 | 1.40 |
| **VHR1** | DNA-binding transcription factor | Nucleus, cytoplasm | 1.56 | 1.35 |
| **VID24** | Subunit of GID (Glucose Induced degradation Deficient) complex | Nucleus, cytoplasm | 2.07 | 1.35 |
| **VRP1** | Actin-binding protein | Cytosol | 1.39 | 1.37 |
| **YPS1** | Aspartic protease | Plasma membrane | 1.89 | 1.38 |

Supplemental Table S3. Overlapping DEGs, 6PPD downregulated, and 6PPD-Q upregulated, with associated functions and organelles. Fold changes are > 1.25 for

upregulated and < -1.25 for downregulated genes. Based on DAVID Bioinformatics Functional Annotation Clustering with enrichment score > 2.0, sulfur metabolism, amino acid biosynthesis, and transport are the main categories downregulated by 6PPD and upregulated by 6PPD-Q.

| Gene | Key Functions | Associated Organelle | 6PPD | 6PPD-Q |
|---|---|---|---|---|
| | | | Fold Change | |
| **ADE3** | Formate-tetrahydrofolate ligase, methylenetetrahydrofolate cycolhydrolase, and methylenetetrahydrofolate dehydrogenase | Nucleus, cytoplasm | -1.26 | 1.26 |
| **ADI1** | Nickel-requiring acireductone dioxygenase | Nucleus, cytoplasm | -1.27 | 1.41 |
| **AGP3** | Amino acid transmembrane transporter | Endoplasmic reticulum | -3.91 | 1.52 |
| **AIM17** | Organization of mitochondrial structure | Mitochondria | -1.73 | 1.74 |
| **ARO10** | Phenylpyruvate decarboxylase | Cytosol | -1.49 | 1.27 |
| **ARO9** | Aromatic-amino-acid:2-oxoglutarate and kynurenine aminotransferase | Nucleus, cytoplasm | -1.34 | 1.32 |
| **BNA3** | L-kynurenine and 2-aminoadipate transaminase | Mitochondria, cytoplasm | -1.99 | 1.53 |
| **CYS3** | Cystathionine gamma-lyase | Nucleus, cytoplasm | -1.54 | 1.26 |
| **DUG2** | Subunit of glutathione hydrolase | Cytoplasm, nuclear periphery | -2.12 | 1.33 |
| **DUG3** | Subunit of glutathione hydrolase | Cytoplasm, nuclear periphery | -2.83 | 1.29 |
| **GCV1** | Glycine cleavage system T-protein | Mitochondria | -1.38 | 1.32 |
| **GCV2** | Mitochondrial glycine decarboxylase | Mitochondria | -1.43 | 1.34 |
| **GLK1** | Glucokinase | Cytosol, plasma membrane | -2.11 | 1.54 |
| **GSY1** | Glycogen synthase | Cytoplasm, mitochondria | -1.48 | 4.30 |
| **GSY2** | Glycogen synthase | Cytoplasm, nucleus | -1.31 | 1.95 |
| **GTO1** | Glutathione transferase | Peroxisome | -1.59 | 1.69 |
| **HSP104** | Adenosine-binding protein chaperone | Nucleus, cytoplasm, endoplasmic reticulum | -1.29 | 1.46 |
| **HSP42** | Putative chaperone | Cytoskeleton | -1.56 | 1.94 |
| **HXK1** | Hexokinase | Cytoplasm, mitochondria | -1.90 | 2.25 |

| | | | | |
|---|---|---|---|---|
| **HXT4** | Glucose and hexose transmembrane transporter | Plasma membrane, cell periphery | -1.98 | 1.81 |
| **HXT7** | Glucose transporter | Plasma membrane | -5.61 | 2.55 |
| **MET1** | Uroporphyrin-III C-methyltransferase | Cytosol, nucleus | -5.55 | 1.92 |
| **MET10** | Alpha subunit of sulfite reductase complex | Cytosol | -2.15 | 1.85 |
| **MET13** | Methylenetetrahydrofolate reductase | Mitochondria | -2.15 | 1.36 |
| **MET14** | ATP sulfurylase | Cytoplasm | -1.99 | 1.66 |
| **MET2** | L-homoserine-O-acetyltransferase | Cytoplasm | -3.80 | 1.80 |
| **MET28** | Transcription activator | Nucleus | -6.45 | 1.76 |
| **MET3** | ATP sulfurylase | Cytoplasm, mitochondria | -3.50 | 1.71 |
| **MET30** | Ubiquitin binding protein | Nucleus | -1.40 | 1.33 |
| **MET32** | Zinc-finger DNA-binding transcription factor | Cytoplasm, nucleus | -2.53 | 2.13 |
| **MET5** | H2S-NADP oxidoreductase | Cytoplasm, vacuole, cell periphery | -3.51 | 1.65 |
| **MET6** | Methionine synthase | Cytoplasm, plasma membrane | -2.43 | 1.36 |
| **MET8** | Precorrin-2 dehydrogenase and sirohydrochlorin ferrochelatase | Cytosol | -2.01 | 1.64 |
| **MHT1** | S-adenosylmethionine-homocysteine S-methyltransferase | Cytoplasm | -3.63 | 1.78 |
| **MMP1** | Transmembrane transporter of S-methylmethionine | Plasma membrane, cell periphery | -3.53 | 1.66 |
| **MNN4** | Enzyme activator protein | Golgi apparatus | -1.45 | 1.26 |
| **MUP1** | Methionine permease | Plasma membrane | -4.47 | 1.46 |
| **MUP3** | L-methionine transporter | Plasma membrane, vacuole | -6.11 | 1.66 |
| **MXR1** | Peptide-methionine (S)-S-oxide reductase | Nucleus, cytoplasm | -1.38 | 1.64 |
| **NDI1** | NADH:ubiquinone oxidoreductase | Mitochondrial matrix | -1.27 | 1.30 |
| **OPT1** | Oligopeptide transmembrane transporter | Plasma membrane | -1.77 | 1.25 |
| **PEX2** | Peroxisomal membrane ubiquitin-protein ligase subunit of the peroxisomal protein import machinery | Peroxisome | -1.39 | 1.34 |
| **PIC2** | Copper ion and inorganic phosphate transmembrane transporter | Mitochondria | -1.73 | 1.59 |

| | | | | |
|---|---|---|---|---|
| **PNC1** | Nicotinamidase | Nucleus, cytoplasm, peroxisome | -1.25 | 1.47 |
| **RAD59** | Double-strand break DNA repair and mitochondrial DNA repair | Nucleus, mitochondrial matrix | -1.97 | 1.75 |
| **RGI1** | Energy reserve metabolism | Nucleus, cytoplasm, cell periphery | -1.83 | 3.49 |
| **RMD6** | UNKNOWN | UNKNOWN | -2.12 | 1.56 |
| **SEO1** | Dipeptide transmembrane transporter | Plasma membrane | -9.37 | 2.19 |
| **SER33** | 3-phosphoglycerate dehydrogenase | Cytoplasm | -2.24 | 1.58 |
| **SOL4** | 6-phosphogluconolactonase | Nucleus, cytoplasm | -1.84 | 1.96 |
| **SUL1** | Sulfate transmembrane transporter | Plasma membrane | -2.06 | 2.89 |
| **SUL2** | Sulfate transmembrane transporter | Plasma membrane | -3.25 | 2.29 |
| **TPK1** | cAMP-dependent protein kinase | Nucleus, cytoplasm | -1.45 | 1.69 |
| **VHT1** | Biotin transmembrane transporter | Plasma membrane | -1.84 | 1.29 |
| **YCT1** | Cysteine transmembrane transporter | Cell periphery, endoplasmic reticulum | -1.55 | 1.72 |
| **ZWF1** | Glucose-6-phosphate dehydrogenase (G6PD) | Cytoplasm, cytosol | -1.33 | 1.45 |

**Supplemental Table S4.** Overlapping DEGs, 6PPD upregulated, and 6PPD-Q downregulated, with associated functions and organelles. Fold changes are > 1.25 for upregulated and < -1.25 for downregulated genes. Based on DAVID Bioinformatics Functional Annotation Clustering with enrichment score > 2.0 there is no trend in dysregulation.

| **Gene** | **Key Functions** | **Associated Organelle** | **6PPD** | **6PPD-Q** |
|---|---|---|---|---|
| | | | **Fold Change** | |
| **ADH7** | Alcohol dehydrogenase | Cytosol | 1.54 | -1.48 |
| **ARG1** | Argininosuccinate synthase | Cytosol | 2.01 | -1.38 |
| **ARG3** | Ornithine carbamoyltransferase | Cytosol | 1.31 | -1.61 |
| **ERG28** | ER-membrane protein | Endoplasmic Reticulum | 1.37 | -1.27 |
| **ERG3** | C-5 sterol desaturase | Endoplasmic Reticulum | 1.36 | -1.33 |
| **PEX21** | Peroxisomal protein transport | Peroxisome | 1.26 | -1.32 |
| **RPL22B** | Subunit of the cytosolic large ribosomal subunit | Ribosome | 1.45 | -1.29 |
| **RPS14B** | rRNA-binding constituent of the cytosolic small ribosomal subunit | Ribosome | 1.36 | -1.29 |
| **SPO21** | Spindle pole body protein | Spindle Body | 1.54 | -1.31 |

| | | | | |
|---|---|---|---|---|
| **SPO24** | Protein involved in sporulation | Prospore Membrane | 1.36 | -1.29 |
